# Insights into non-autoimmune type 1 diabetes with 13 novel loci in low polygenic risk score patients

**DOI:** 10.1038/s41598-021-94994-9

**Published:** 2021-08-06

**Authors:** Jingchun Qu, Hui-Qi Qu, Jonathan P. Bradfield, Joseph T. Glessner, Xiao Chang, Lifeng Tian, Michael March, John J. Connolly, Jeffrey D. Roizen, Patrick M. A. Sleiman, Hakon Hakonarson

**Affiliations:** 1 The Center for Applied Genomics, Children’s Hospital of Philadelphia, 3615 Civic Center Blvd, Abramson Building, Philadelphia, PA 19104 USA; 2 Quantinuum Research LLC, San Diego, CA 92101 USA; 3 Department of Pediatrics, The Perelman School of Medicine, University of Pennsylvania, Philadelphia, PA 19104 USA; 4 Division of Human Genetics, Children’s Hospital of Philadelphia, Philadelphia, PA 19104 USA; 5 Division of Pulmonary Medicine, Children’s Hospital of Philadelphia, Philadelphia, PA 19104 USA

**Keywords:** Genetic association study, Endocrinology

## Abstract

With polygenic risk score (PRS) for autoimmune type 1 diabetes (T1D), this study identified T1D cases with low T1D PRS and searched for susceptibility loci in these cases. Our hypothesis is that genetic effects (likely mediated by relatively rare genetic variants) of non-mainstream (or non-autoimmune) T1D might have been diluted in the previous studies on T1D cases in general. Two cohorts for the PRS modeling and testing respectively were included. The first cohort consisted of 3302 T1D cases and 6181 controls, and the independent second cohort consisted of 3297 T1D cases and 6169 controls. Cases with low T1D PRS were identified using PRSice-2 and compared to controls with low T1D PRS by genome-wide association (GWA) test. Thirteen novel genetic loci with high imputation quality (Quality Score r^2^ > 0.91) were identified of SNPs/SNVs associated with low PRS T1D at genome-wide significance (*P* ≤ 5.0 × E−08), in addition to 4 established T1D loci, 3 reported loci by our previous study, as well as 9 potential novel loci represented by rare SNVs, but with relatively low imputation quality (Quality Score r^2^ < 0.90). For the 13 novel loci, 9 regions have been reported of association with obesity related traits by previous GWA studies. Three loci encoding long intergenic non-protein coding RNAs (lncRNA), and 2 loci involved in N-linked glycosylation are also highlighted in this study.

## Introduction

Type 1 diabetes (T1D) is caused by T-cell mediated autoimmune destruction of pancreatic β-cells^1^. There is no cure for T1D to date. The molecular mechanisms underlying T1D are complex and not completely understood. Human genetic studies have uncovered multiple T1D genes that contribute to our understanding of the pathogenesis ofT1D^2–7^. With the rapid advances in human genomics technology in recent years, over 70 T1D loci have been identified^8^ (https://www.ebi.ac.uk/gwas/). While these discoveries of T1D-associated genes have greatly increased our knowledge of T1D, our current genetic knowledge on T1D is far from complete, and a large number of T1D genes remain uncovered^9^. A key bottleneck for the GWAS approach is limitation of sample size even with the presence of collaborative international consortia^10^. The phenotype of type 1 diabetes has been regarded as heterogeneous. While the majority of T1D patients have autoimmune disease, 5–10% of Caucasian diabetic subjects with recent-onset T1D do not have islet cell antibodies, often referred to as T1bD^11^. Due to different pathogenesis, T1bD cases may be associated with different genetic loci from autoimmune T1D, or T1aD. However, the smaller proportion of T1bD cases suggests that T1bD-related genetic effects have been diluted in the previous studies with T1D cases studied in general. Besides T1bD, the non-autoimmune and monogenic form of pediatric diabetes, maturity-onset diabetes of the young (MODY) cases, may be misdiagnosed as T1D^12^, which further contributes to the heterogeneity of the T1D phenotype.

With numerous genetic loci for many human complex diseases identified to date, polygenic risk scores (PRS) aggregate the effects of many genetic variants across the human genome into a single score, an approach that has been shown of improve disease prediction and differential diagnosis^13^. The T1D loci identified by the GWAS studies to date are mainly associated with the genetic susceptibility of the major component of the heterogeneous T1D phenotype, i.e. T1aD, while the genetic susceptibility of the minor non-autoimmune components (e.g. T1bD and misdiagnosed MODY) are under-represented in those results likely as a result of being diluted. In this study, we propose that a high T1D PRS score predicts or suggests a T1aD case, whereas a low T1D PRS score in a T1D case suggests the opposite and represents our major interest in this study. Our aim in this study is to identify low PRS T1D cases and to run a separate GWAS in an attempt to uncover genetic loci associated with T1bD patients. Our approach effectively concentrates the dilution of non-mainstream T1D by excluding high PRS T1D cases, to uncover novel genetic loci associated with non-mainstream T1D. Therefore, the dilution of low PRS T1D by misdiagnosed MODY is not a concern. On the other hand, although the low PRS cases may include MODY patients, there are no MODY mutation identified with genome-wide significance in this GWAS study, which is as expected while next generation sequencing, e.g. whole exome sequencing, is the more proper approach.

## Methods

### Subjects

6599 European T1D cases and 12,350 European controls were included in this study. The T1D cases were from the Children's Hospital of Philadelphia (CHOP)^14^, The Montreal Children's Hospital^14^, The Diabetes Control and Complications Trial—Epidemiology of Diabetes Interventions and Complications (DCCT-EDIC) cohort (http://www.ncbi.nlm.nih.gov/projects/gap/cgi-bin/study.cgi?study_id=phs000086.v2.p1), the Type 1 Diabetes Genetics Consortium (T1DGC, http://www.ncbi.nlm.nih.gov/projects/gap/cgi-bin/study.cgi?study_id=phs000180.v1.p1), and later recruited subjects at CHOP, respectively. The T1D cases were mainly recruited by clinical diagnosis, i.e. insulin dependent for at least 6 months, and diagnosed under the age of 18 for the subjects recruited at CHOP and Montreal. The non-mainstream T1D cases in this study were defined by low T1D PRS scores, with the cut-off value of PRC determined by Receiver Operating Characteristic (ROC) curve analysis. The included cases were all confirmed of European ancestry by principal component analysis (PCA) with genome-wide SNP markers, with individuals from other populations or with admixed ancestries excluded. The genotyping was done by the Illumina Human Hap550 Genotyping BeadChip or a newer version of Illumina Genotyping BeadChip. Other demographic, phenotypic and genotypic details about these individuals were described in our previous publication^15^. Imputation of single nucleotide polymorphisms (SNP) on auto-chromosomes was done using the TOPMed Imputation Server (https://imputation.biodatacatalyst.nhlbi.nih.gov) with the TOPMed (Version R2 on GRC38) Reference Panel, with the quality filters of R^2^ ≥ 0.3. Altogether, 104,689,647 autosomal single nucleotide variants (SNV) with quality R^2^ ≥ 0.3 were included in this study. Population stratification was assessed by PCA analysis, and genetic association tests conditioned on sex were corrected by the first 10 principal components (PC). The association test was done using PLINK1.9 software^16^.

### Polygenic risk scores (PRS)

To avoid the issue of overfitting for PRS scoring, the subjects were randomly split into two independent cohorts without duplication, i.e. the PRS training cohort (Cohort A) including 3302 T1D cases (1739 males, 1560 females, and 3 cases with undetermined sex) and 6181 controls(3326 males, 2840 females, and 15 cases with undetermined sex), and the PRS testing cohort (Cohort B) including 3297 T1D cases (1744 males, 1549 females, and 4 cases with undetermined sex) and 6169 controls (3339 males, 2818 females, and 12 cases with undetermined sex). PRSs of the test cohort were calculated using the Polygenic Risk Score software (PRSice-2)^17^, based on the statistics of the training group. The performance of a series of cutoff of T1D association *P* values (including 10^–10^, 10^–9^ , 10^–8^, 10^–7^, 10^–6^, 10^–5^, 10^–4^, 0.001, 0.01, 0.05, 0.1, 0.2, and 1) for selection of SNP markers was assessed by the Area Under the ROC Curve (AUC). The *P* value cutoff with the largest AUC was adopted.

### GWAS of T1D patients with low PRS

**Figure 1:**
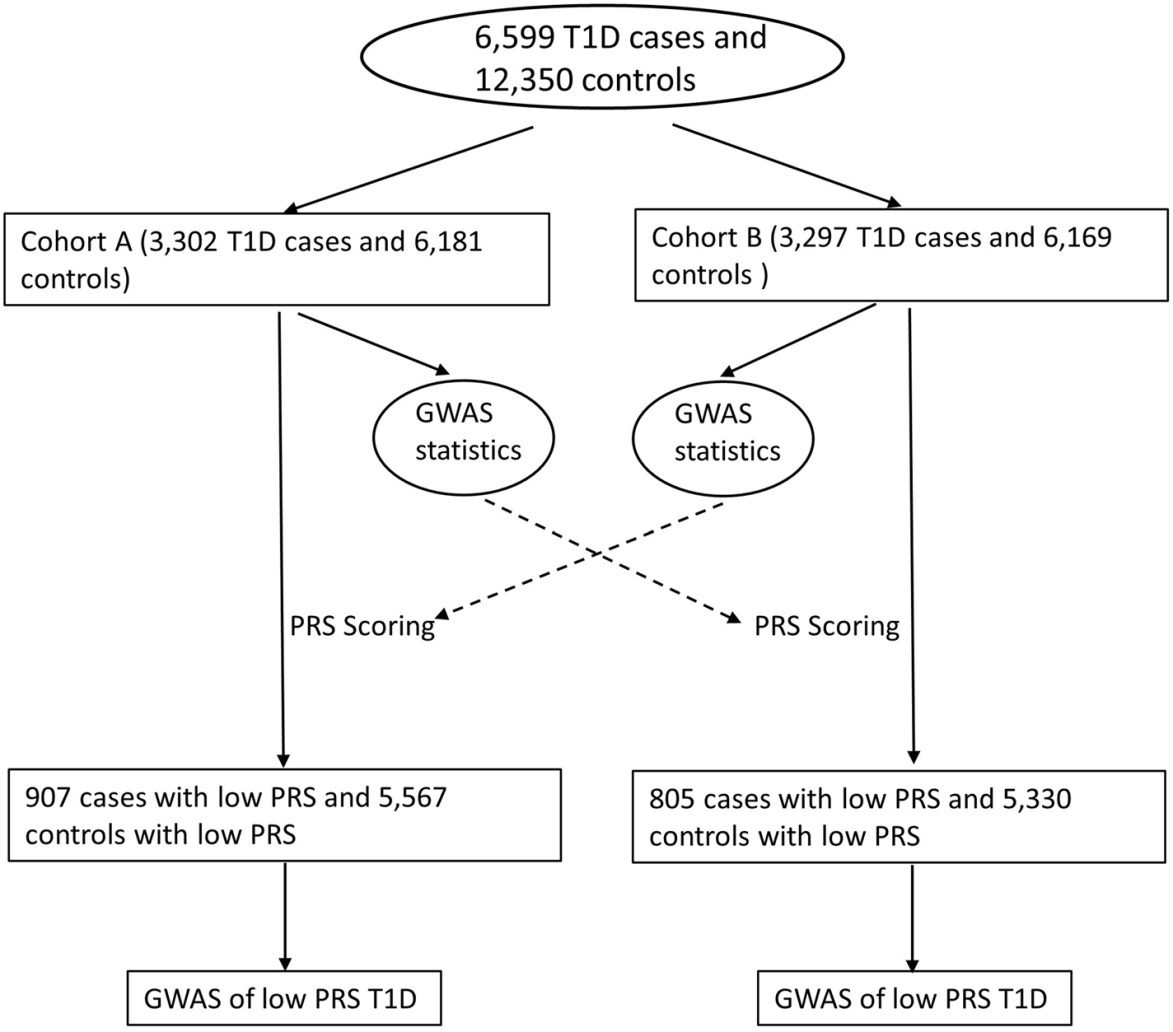
The flow chart of the study approach.

The flow chart of the study approach is shown in Fig. 1. According to the PRS values, the T1D patients were separated into two groups, i.e. a low PRS group and a high PRS group. The PRS cutoff was determined by the maximum Matthews correlation coefficient (MCC). Using the same PRS cutoff, health controls with low T1D PRS were identified. The GWAS of T1D patients with low PRS was performed by comparing to health controls with low T1D PRS. The Manhattan plots were done using the SNPEVG software^18^. Genetic association signals within each locus were plotted by LocusZoom^19^.

### Cohort switch

Consequently, we switched the two cohorts, i.e. using Cohort B for the statistics of PRS modelling, then we tested the PRS models in Cohort A. GWAS of T1D patients with low PRS was done using the same approach as described above.

### Data and resource availability

The datasets generated during and/or analyzed during the current study are available from the corresponding author upon reasonable request.

All methods were carried out in accordance with relevant guidelines and regulations. The study was approved by the Institutional Review Boards of Children’s Hospital of Philadelphia (CHOP). Written informed consent was obtained from each participating subject or, if subjects are under 18, their parent/guardian.

### Significance statement

Type 1 diabetes (T1D) is a highly heterogeneous genetic disease. Human genetic and genomic study on T1D has gained us significant knowledge on the molecular basis of autoimmunity in T1D. However, it has been recognized for long that a small number of T1D cases present without autoantibodies and are considered non-autoimmune. Human genetic approach has not been helpful for the study of these patients, as genetic effects of these non-mainstream (or non-autoimmune) T1D have been diluted in the previous studies on T1D cases in general. For the first, we identified non-mainstream T1D cases represented by low T1D polygenic risk score (PRS), and identified 13 novel loci represented by rare SNVs. This study presents a brand-new genomic landscape of pediatric T1D.

## Results

### AUC of different cutoffs of T1D association *P* values for SNP selection and PRS

The AUCs of different cutoffs of T1D association *P* values for selection of SNP sets are shown in Table 1a. The best AUC (0.8607) is seen at the cutoff of *P* value ≤ 1E−05, which suggests that stricter cutoff may cause the missing of informative SNPs, while looser may introduce noise by including SNPs with spurious T1D association. Based on the SNP markers with T1D association *P* value ≤ 1E−05, a PRS score was acquired for each individual in the independent test cohort. By the maximum MCC (Supplementary Table 1), a PRS cutoff of 1.11E−03 has the maximum MCC (0.6294). A PRS ≤ 1.11E−03 was defined as low risk, and a PRS > 1.11E−03 was defined as high risk. With this threshold, the sensitivity (True positive rate, TPR) for T1D prediction is 75.9%, and the specificity (True negative rate, TFR) for T1D prediction is 86.4%. By PRS ≤ 1.11E−03, 805 (24.4%, including 407 males, 396 females, and 2 cases with undetermined sex) out of 3297 T1D cases had low PRS; and 5330 (86.4%, including 2882 males, 2436 females, and 12 cases with undetermined sex) out of 6169 controls had low PRS.

**Table 1:** The AUCs of different cutoffs of T1D association *P* values.

*P* value*	AUC**
**a. First cohort**	
≤ 1.00E−10	0.8462
≤ 1.00E−09	0.8487
≤ 1.00E−08	0.8518
≤ 1.00E−07	0.8565
≤ 1.00E−06	0.8604
≤ 1.00E−05	0.8607
≤ 1.00E−04	0.8590
≤ 0.001	0.8561
≤ 0.01	0.8546
≤ 0.05	0.8502
≤ 0.1	0.8508
≤ 0.2	0.8530
≤ 0.5	0.8563
≤ 1	0.8579
**b. Switched cohort**	
≤ 1.00E−10	0.8576
≤ 1.00E−09	0.8589
≤ 1.00E−08	0.8588
≤ 1.00E−07	0.8609
≤ 1.00E−06	0.8633
≤ 1.00E−05	0.8654
≤ 1.00E−04	0.8618
≤ 0.001	0.8555
≤ 0.01	0.8470
≤ 0.05	0.8441
≤ 0.1	0.8446
≤ 0.2	0.8467
≤ 0.5	0.8521
≤ 1	0.8533

*The *P* values are based on the statistics of the PRS training cohort.

**The AUCs are the PRS performances in the independent testing cohort.

### GWAS of T1D patients with low PRS

**Figure 2:**
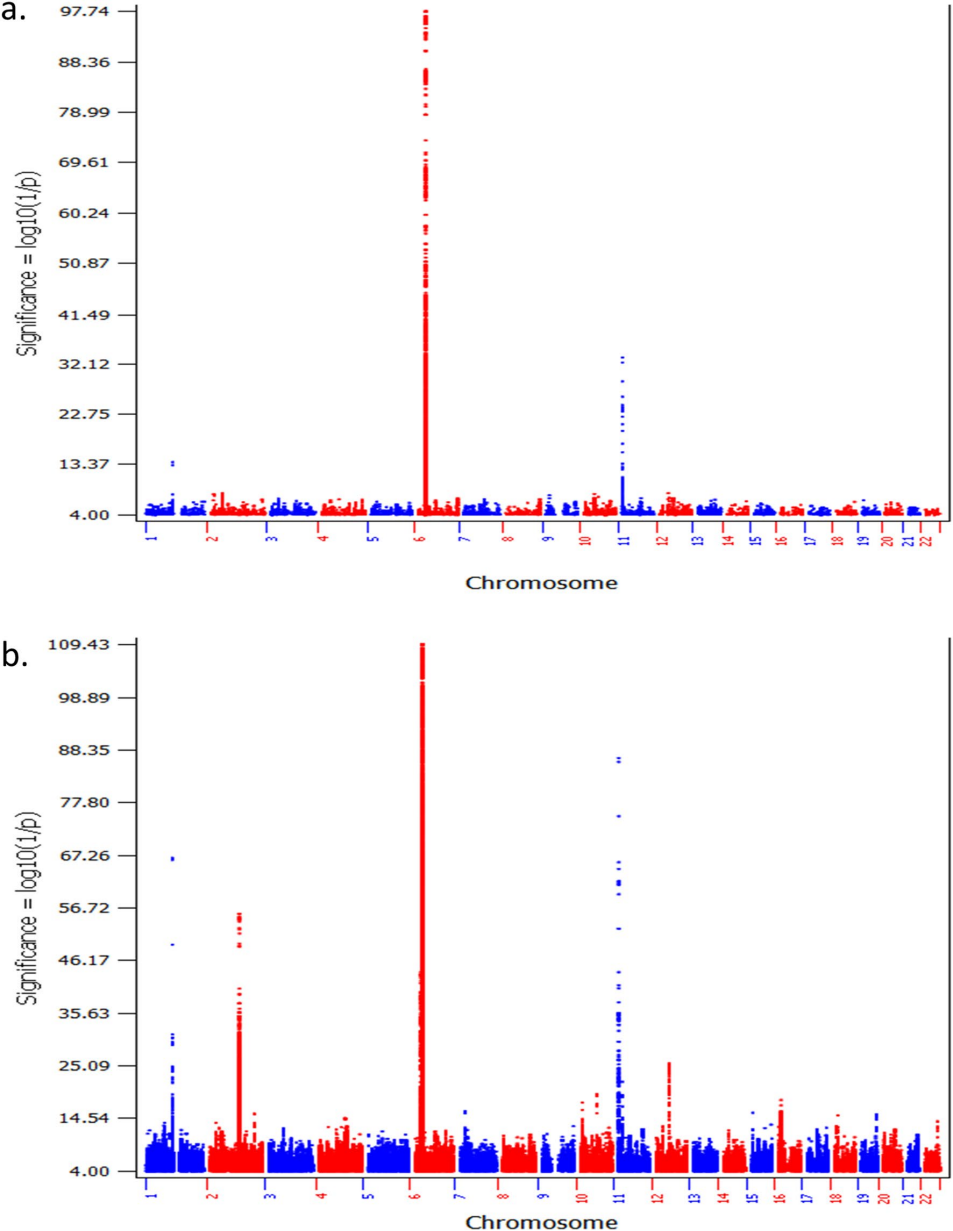
The Manhattan plots of cohort B. (**a**) The plot of the GWAS of T1D patients with low T1D PRS compared to controls with low T1D PRS (805 cases vs. 5330 controls); (**b**) the plot of the GWAS of all T1D patients compared to all controls (3297 cases vs. 6169 controls).

The GWAS of T1D patients with low T1D PRS compared to controls with low T1D PRS identified a large number of SNPs associated with T1D with genome-wide significance (*P* ≤ 5.0 × E−08), from 10 genetic loci (Supplementary Table 2, Fig. 2). Among these 10 genetic loci, 3 loci have been established of T1D association by previous studies, including *HLA*, *INS*, and *PTPN22* (Table 2a). By looking at the established leading T1D signal of each locus, the frequencies of the predisposing alleles of *HLA* and *PTPN22* were lower in the low T1D PRS cohort, while the protective allele of *INS* were higher in the low T1D PRS cohort. The effect sizes of *HLA* (*P* = 6.67E−08) and *PTPN22* (*P* = 0.052) were smaller in the low PRS cases. Besides these 3 established T1D loci, 7 loci associated with low PRS T1D were identified (Table 3a). LocusZoom plots for genetic association signals within each locus are shown in Supplementary Figures 1–7. The association signals of these loci are only seen in low PRS T1D cases, but not in the T1D cases overall, and were missed previously due to diluted genetic effects. Among the 7 loci, 6 loci are novel, while the ankyrin 3 (*ANK3*) locus, related to neural control of the endocrine pancreas^20^, has been identified of genome-wide significance in our study on low T1D genetic risk scores (GRS) patients^21,22^.

**Table 2:** Leading SNPs at three loci have been established of T1D association.

CHR	dbSNP	BP (hg38)	Gene.refGene	A1	Quality Score (r^2^)	Genotyped	Low PRS cases versus low PRS controls				U95	P	All cases versus all controls in cohort B				U95	*P*	OR heterogeneity *P*
							MAF	*n*	OR	L95			MAF	*n*	OR	L95			
**a. First cohort**																			
1	rs2476601	11,38,34,946	PTPN22	A	0.99897	Genotyped	0.08676	6118	1.87	1.594	2.194	1.55E−14	0.1112	9450	2.244	2.048	2.458	1.92E−67	0.052
6	rs9273368	3,26,58,698	HLA-DQB1	A	0.97908	Imputed	0.2586	6118	4.073	3.616	4.587	1.24E−118	0.3841	9450	6.018	5.572	6.5	< *1E*−*350*	6.67E−08
11	rs689	21,60,994	INS	A	0.89726	Imputed	0.2572	6118	0.391	0.3354	0.4559	4.02E−33	0.2331	9450	0.4442	0.4098	0.4815	1.03E−86	0.149
**b. Switched cohort**																			
1	rs2476601	11,38,34,946	PTPN22	A	0.99897	Genotyped	0.09515	6460	2.216	1.921	2.557	1.03E−27	0.1152	9465	2.268	2.07	2.484	3.53E−69	0.789
6	rs9273368	3,26,58,698	HLA-DQB1	A	0.97908	Imputed	0.2584	6460	3.809	3.416	4.247	6.19E−128	0.376	9465	5.937	5.503	6.404	< *1E*−*350*	5.49E−11
11	rs689	21,60,994	INS	A	0.89726	Imputed	0.259	6460	0.4553	0.3972	0.5218	1.23E−29	0.2366	9465	0.4883	0.4517	0.5279	1.54E−72	0.383
12	rs1702877	5,60,34,024	IKZF4	T	0.98704	Imputed	0.3276	6460	1.353	1.22	1.501	1.03E−08	0.3427	9465	1.366	1.282	1.455	3.59E−22	0.877

Italic values indicate the smallest *P* value.

**Table 3:** Novel loci associated with low PRS T1D.

CHR	BP (hg38)	SNP	dbSNP	allele	allele_Frq	Quality Score (r^2^)	Genotyped	n	OR	L95	U95	*P*	n	OR	L95	U95	*P*	Func.refGene	Gene.refGene
								Low PRS cases versus low PRS controls					All cases versus all controls in the cohort						
**a. First cohort**																			
2	1,07,27,877	chr2:10727877:C:T	rs147458998	T	0.00203	0.718	Imputed	6118	11.06	4.818	25.4	1.45E−08	9450	3.201	1.508	6.795	0.002454	Intronic	ATP6V1C2
2	1,71,26,012	chr2:17126012:C:T	rs56806432	T	0.03445	0.97878	Imputed	6118	1.936	1.539	2.437	1.74E−08	9450	1.149	0.976	1.352	0.09531	Intergenic	FAM49A;RAD51AP2
2	1,71,59,712	chr2:17159712:C:A	rs16983255	A	0.03454	0.97987	Imputed	6118	1.916	1.523	2.411	2.85E−08	9450	1.142	0.9704	1.343	0.1101	Intergenic	FAM49A;RAD51AP2
2	1,71,59,927	chr2:17159927:C:T	rs60975145	T	0.03448	0.9799	Imputed	6118	1.927	1.532	2.425	2.17E−08	9450	1.148	0.9755	1.351	0.09667	Intergenic	FAM49A;RAD51AP2
2	5,15,13,429	chr2:51513429:G:A	rs28958299	A	0.00609	0.98423	Imputed	6118	3.4	2.194	5.269	4.39E−08	9450	1.527	1.05	2.223	0.02693	ncRNA_intronic	LOC730100
2	5,14,87,374	chr2:51487374:C:CA		CA	0.0063	0.98739	Imputed	6118	3.415	2.201	5.298	4.25E−08	9450	1.566	1.079	2.275	0.0184		
2	5,15,04,453	chr2:51504453:C:T	rs57623361	T	0.00667	0.98795	Imputed	6118	3.251	2.129	4.964	4.75E−08	9450	1.532	1.07	2.192	0.0198	ncRNA_intronic	LOC730100
2	5,15,31,113	chr2:51531113:T:A	rs28957091	A	0.00679	0.98915	Imputed	6118	3.408	2.239	5.188	1.07E−08	9450	1.611	1.124	2.308	0.009372	ncRNA_intronic	LOC730100
2	5,15,28,593	chr2:51528593:T:C	rs1406418	C	0.00716	0.99126	Imputed	6118	3.166	2.094	4.789	4.74E−08	9450	1.531	1.079	2.172	0.01703	ncRNA_intronic	LOC730100
2	5,15,30,016	chr2:51530016:T:G	rs28958318	G	0.0073	0.99201	Imputed	6118	3.205	2.122	4.84	3.10E−08	9450	1.579	1.113	2.24	0.01039	ncRNA_intronic	LOC730100
2	5,15,20,595	chr2:51520595:C:T	rs28957085	T	0.00601	0.99376	Imputed	6118	3.575	2.31	5.533	1.09E−08	9450	1.616	1.113	2.346	0.01167	ncRNA_intronic	LOC730100
2	5,14,86,782	chr2:51486782:AG:A		A	0.00593	0.99402	Imputed	6118	3.445	2.213	5.364	4.30E−08	9450	1.476	1.01	2.156	0.04423	ncRNA_intronic	LOC730100
2	5,15,25,890	chr2:51525890:A:G	rs1528792	G	0.00684	0.99484	Imputed	6118	3.27	2.159	4.951	2.18E−08	9450	1.529	1.075	2.174	0.01814	ncRNA_intronic	LOC730100
2	5,15,28,054	chr2:51528054:T:C	rs28957087	C	0.00735	0.99609	Imputed	6118	3.306	2.195	4.979	1.04E−08	9450	1.607	1.135	2.276	0.007525	ncRNA_intronic	LOC730100
9	1,42,29,050	chr9:14229050:G:C	rs10961435	C	0.00389	0.95755	Imputed	6118	4.762	2.753	8.237	2.39E−08	9450	1.806	1.103	2.957	0.01885	Intronic	NFIB
10	4,42,60,591	chr10:44260591:C:T	rs746298	T	0.00446	0.98941	Imputed	6118	4.175	2.552	6.83	1.26E−08	9450	2.304	1.485	3.576	0.000197	Intergenic	LINC00841;C10orf142
10	6,06,64,647	chr10:60664647:T:C	rs1816797	C	0.00183	0.93498	Imputed	6118	8.974	4.087	19.7	4.55E−08	9450	4.188	2.04	8.599	9.52E−05	Intronic	ANK3
12	3,46,99,338	chr12:34699338:G:T	rs12424461	T	0.00231	0.85309	Imputed	6118	8.528	4.101	17.73	9.54E−09	9450	2.127	1.105	4.094	0.02396	Intergenic	ALG10;NONE
**b. Switched cohort**																			
2	6,24,48,472	chr2:62448472:C:T	rs78389245	T	0.00223	0.93017	Imputed	6460	6.453	3.353	12.42	2.37E−08	9465	2.246	1.248	4.041	0.006953	Intergenic	B3GNT2;TMEM17
2	6,24,42,411	chr2:62442411:A:G	rs75610843	G	0.00349	0.96936	Imputed	6460	5.023	2.83	8.917	3.54E−08	9465	2.114	1.278	3.498	0.003576	Intergenic	B3GNT2;TMEM17
2	6,24,46,036	chr2:62446036:C:T	rs76505469	T	0.0031	0.96962	Imputed	6460	5.655	3.117	10.26	1.20E−08	9465	1.959	1.156	3.319	0.01245	Intergenic	B3GNT2;TMEM17
2	6,24,40,366	chr2:62440366:C:T	rs75921605	T	0.00346	0.97057	Imputed	6460	5.023	2.83	8.917	3.54E−08	9465	2.114	1.278	3.498	0.003576	Intergenic	B3GNT2;TMEM17
2	7,03,52,234	chr2:70352234:A:G	rs116081627	G	0.002	0.91477	Imputed	6460	7.679	3.755	15.7	2.34E−08	9465	1.911	1	3.651	0.05001	Intergenic	FAM136A;TGFA
3	4,89,51,134	chr3:48951134:T:C	rs143836109	C	0.00397	0.58298	Imputed	6460	4.986	2.801	8.875	4.77E−08	9465	1.782	1.059	2.999	0.02969	Intronic	ARIH2
4	8,83,57,077	chr4:88357077:T:C	rs76377119	C	0.002	0.71507	Imputed	6460	9.545	4.389	20.76	1.26E−08	9465	4.076	1.927	8.622	0.000237	Intergenic	LOC105369192;HERC6
4	14,15,56,946	chr4:141556946:C:T	rs72615940	T	0.00275	0.95975	Imputed	6460	6.361	3.301	12.26	3.25E−08	9465	2.651	1.469	4.784	0.001204	Intergenic	LINC02432;IL15
4	14,15,57,792	chr4:141557792:T:C	rs115762557	C	0.00279	0.96694	Imputed	6460	6.046	3.166	11.54	4.96E−08	9465	2.474	1.368	4.475	0.002725	Intergenic	LINC02432;IL15
4	14,15,58,035	chr4:141558035:A:C	rs72615945	C	0.00278	0.96769	Imputed	6460	6.046	3.166	11.54	4.96E−08	9465	2.474	1.368	4.475	0.002725	Intergenic	LINC02432;IL15
4	14,15,58,497	chr4:141558497:G:GATTTTCA		GATTTTCA	0.00278	0.96769	Imputed	6460	6.046	3.166	11.54	4.96E−08	9465	2.474	1.368	4.475	0.002725		
4	14,15,58,842	chr4:141558842:C:T	rs72615950	T	0.00278	0.96771	Imputed	6460	6.046	3.166	11.54	4.96E−08	9465	2.474	1.368	4.475	0.002725	Intergenic	LINC02432;IL15
4	14,15,59,253	chr4:141559253:G:A	rs72615951	A	0.00278	0.96771	Imputed	6460	6.046	3.166	11.54	4.96E−08	9465	2.474	1.368	4.475	0.002725	Intergenic	LINC02432;IL15
4	14,15,59,260	chr4:141559260:G:A	rs72615952	A	0.00278	0.96771	Imputed	6460	6.046	3.166	11.54	4.96E−08	9465	2.474	1.368	4.475	0.002725	Intergenic	LINC02432;IL15
4	14,15,57,845	chr4:141557845:T:A	rs77083164	A	0.00278	0.96773	Imputed	6460	6.046	3.166	11.54	4.96E−08	9465	2.474	1.368	4.475	0.002725	Intergenic	LINC02432;IL15
4	14,15,57,678	chr4:141557678:A:G	rs75736694	G	0.00278	0.96774	Imputed	6460	6.046	3.166	11.54	4.96E−08	9465	2.474	1.368	4.475	0.002725	Intergenic	LINC02432;IL15
4	14,15,58,749	chr4:141558749:G:A	rs72615948	A	0.00278	0.96774	Imputed	6460	6.046	3.166	11.54	4.96E−08	9465	2.474	1.368	4.475	0.002725	Intergenic	LINC02432;IL15
4	14,15,57,600	chr4:141557600:A:G	rs72615942	G	0.00278	0.96775	Imputed	6460	6.046	3.166	11.54	4.96E−08	9465	2.474	1.368	4.475	0.002725	Intergenic	LINC02432;IL15
4	14,15,58,193	chr4:141558193:G:C	rs72615946	C	0.00278	0.96775	Imputed	6460	6.046	3.166	11.54	4.96E−08	9465	2.474	1.368	4.475	0.002725	Intergenic	LINC02432;IL15
4	14,15,58,199	chr4:141558199:C:T	rs72615947	T	0.00278	0.96775	Imputed	6460	6.046	3.166	11.54	4.96E−08	9465	2.474	1.368	4.475	0.002725	Intergenic	LINC02432;IL15
4	14,15,57,291	chr4:141557291:T:C	rs72615941	C	0.00278	0.96776	Imputed	6460	6.046	3.166	11.54	4.96E−08	9465	2.474	1.368	4.475	0.002725	Intergenic	LINC02432;IL15
4	14,15,56,501	chr4:141556501:C:T	rs17007424	T	0.00256	0.97308	Imputed	6460	6.361	3.301	12.26	3.25E−08	9465	2.454	1.35	4.462	0.003252	Intergenic	LINC02432;IL15
4	14,15,56,393	chr4:141556393:A:G	rs72615939	G	0.00257	0.97583	Imputed	6460	6.361	3.301	12.26	3.25E−08	9465	2.454	1.35	4.462	0.003252	Intergenic	LINC02432;IL15
4	14,15,56,881	chr4:141556881:C:T	rs200157898	T	0.00257	0.97584	Imputed	6460	6.361	3.301	12.26	3.25E−08	9465	2.454	1.35	4.462	0.003252	Intergenic	LINC02432;IL15
4	14,15,57,024	chr4:141557024:TACTC:T		T	0.00257	0.97591	Imputed	6460	6.361	3.301	12.26	3.25E−08	9465	2.454	1.35	4.462	0.003252	Intergenic	LINC02432;IL15
5	15,94,80,234	chr5:159480234:C:T	rs117952033	T	0.00138	0.83707	Imputed	6460	14.31	5.602	36.55	2.68E−08	9465	4.707	1.862	11.9	0.001062	Intergenic	LINC01845;LINC01847
5	15,94,99,811	chr5:159499811:C:T	rs10515798	T	0.00148	0.84382	Imputed	6460	14.31	5.602	36.55	2.68E−08	9465	4.707	1.862	11.9	0.001062	Intergenic	LINC01845;LINC01847
6	17,02,83,857	chr6:170283857:C:T	rs3734776	T	0.00695	0.93794	Imputed	6460	3.602	2.351	5.518	3.87E−09	9465	1.912	1.334	2.741	0.000422	Exonic	DLL1
6	17,02,88,217	chr6:170288217:C:T	rs3818115	T	0.00634	0.93938	Imputed	6460	3.633	2.336	5.649	1.02E−08	9465	1.819	1.255	2.638	0.001589	Intronic	DLL1
6	17,02,84,744	chr6:170284744:C:T	rs2273214	T	0.00632	0.94094	Imputed	6460	3.633	2.336	5.649	1.02E−08	9465	1.819	1.255	2.638	0.001589	Intronic	DLL1
6	17,02,90,297	chr6:170290297:C:T	rs3823301	T	0.00644	0.94127	Imputed	6460	3.639	2.356	5.619	5.74E−09	9465	1.791	1.241	2.585	0.001843	UTR5	DLL1
6	17,02,87,112	chr6:170287112:A:C	rs3800238	C	0.00632	0.94131	Imputed	6460	3.633	2.336	5.649	1.02E−08	9465	1.819	1.255	2.638	0.001589	Intronic	DLL1
6	17,02,87,178	chr6:170287178:C:A	rs3800237	A	0.00861	0.94352	Imputed	6460	3.085	2.12	4.49	3.98E−09	9465	1.543	1.126	2.115	0.007003	Intronic	DLL1
7	7,20,10,707	chr7:72010707:G:C	rs118182411	C	0.00376	0.93903	Imputed	6460	4.074	2.474	6.71	3.42E−08	9465	1.513	0.9562	2.393	0.07696	Intronic	CALN1
7	8,93,18,777	chr7:89318777:C:T	rs77205087	T	0.00582	0.9747	Imputed	6460	3.447	2.238	5.308	1.94E−08	9465	1.321	0.8943	1.951	0.1619	Intronic	ZNF804B
7	8,93,09,079	chr7:89309079:C:T	rs76060515	T	0.00578	0.97724	Imputed	6460	3.447	2.238	5.308	1.94E−08	9465	1.321	0.8943	1.951	0.1619	Intronic	ZNF804B
7	10,08,14,253	chr7:100814253:G:A	rs3890144	A	0.00757	0.95297	Imputed	6460	3.061	2.066	4.537	2.47E−08	9465	1.352	0.959	1.906	0.0853	Intronic	EPHB4
7	10,07,99,808	chr7:100799808:C:T	rs60224425	T	0.00756	0.95354	Imputed	6460	3.021	2.043	4.467	3.01E−08	9465	1.403	1	1.966	0.04969	Intergenic	ZAN;EPHB4
9	11,02,69,080	chr9:110269080:T:G	rs10816957	G	0.00712	0.94616	Imputed	6460	3.054	2.063	4.52	2.44E−08	9465	1.306	0.9282	1.838	0.1254	Intergenic	TXN;TXNDC8
12	3,36,97,680	chr12:33697680:T:C	rs11052850	C	0.00691	0.99026	Imputed	6460	2.758	1.918	3.966	4.37E−08	9465	1.131	0.8173	1.565	0.4581	Intergenic	SYT10;ALG10
12	3,36,97,996	chr12:33697996:G:A	rs1352395	A	0.0069	0.9906	Imputed	6460	2.758	1.918	3.966	4.37E−08	9465	1.131	0.8173	1.565	0.4581	Intergenic	SYT10;ALG10
12	3,36,95,324	chr12:33695324:G:A	rs12228218	A	0.00689	0.9914	Imputed	6460	2.758	1.918	3.966	4.37E−08	9465	1.131	0.8173	1.565	0.4581	Intergenic	SYT10;ALG10
12	3,36,80,534	chr12:33680534:G:A	rs4142676	A	0.00691	0.99149	Imputed	6460	2.758	1.918	3.966	4.37E−08	9465	1.131	0.8173	1.565	0.4581	Intergenic	SYT10;ALG10
12	3,36,93,014	chr12:33693014:T:C	rs11052843	C	0.00689	0.99193	Imputed	6460	2.758	1.918	3.966	4.37E−08	9465	1.131	0.8173	1.565	0.4581	Intergenic	SYT10;ALG10
12	3,36,97,208	chr12:33697208:T:C	rs2087269	C	0.00689	0.992	Imputed	6460	2.758	1.918	3.966	4.37E−08	9465	1.131	0.8173	1.565	0.4581	Intergenic	SYT10;ALG10
12	3,36,95,992	chr12:33695992:G:T	rs11052847	T	0.00689	0.99201	Imputed	6460	2.758	1.918	3.966	4.37E−08	9465	1.131	0.8173	1.565	0.4581	Intergenic	SYT10;ALG10
12	13,29,15,197	chr12:132915197:G:A	rs11147161	A	0.00503	0.96369	Imputed	6460	3.768	2.344	6.059	4.36E−08	9465	1.366	0.8825	2.115	0.1617	Intergenic	LOC101928530;ZNF605
12	13,28,98,032	chr12:132898032:A:C	rs12230138	C	0.00501	0.96567	Imputed	6460	3.899	2.436	6.239	1.41E−08	9465	1.411	0.9143	2.177	0.1199	Intergenic	LOC101928530;ZNF605
15	6,97,78,062	chr15:69778062:T:C	rs75516437	C	0.00621	0.64954	Imputed	6460	3.937	2.498	6.207	3.60E−09	9465	1.559	1.033	2.353	0.03452	Intergenic	PCAT29;LINC00593
15	9,23,50,924	chr15:92350924:T:C		T	0.02086	0.30905	Imputed	6460	3.36	2.177	5.186	4.45E−08	9465	1.432	0.9822	2.089	0.06196	Intergenic	SLCO3A1;ST8SIA2
20	5,50,80,179	chr20:55080179:G:A	rs144851111	A	0.00386	0.30246	Imputed	6460	8.283	4.084	16.8	4.62E−09	9465	2.649	1.352	5.191	0.00452	Intergenic	DOK5;LINC01441
21	2,81,70,508	chr21:28170508:A:C	rs2831598	C	0.00548	0.85895	Imputed	6460	3.812	2.421	6.001	7.48E−09	9465	1.972	1.34	2.903	0.000575	ncRNA_intronic	LINC01695
21	2,81,64,783	chr21:28164783:C:T	rs139600098	T	0.00601	0.86242	Imputed	6460	3.641	2.37	5.594	3.69E−09	9465	1.825	1.265	2.631	0.001282	ncRNA_intronic	LINC01695
21	2,84,29,678	chr21:28429678:G:A	rs145901638	A	0.0053	0.8642	Imputed	6460	4.115	2.594	6.527	1.86E−09	9465	1.829	1.229	2.722	0.002898	Intergenic	LINC01695;LINC00161
21	2,81,65,396	chr21:28165396:C:A	rs140035545	A	0.00619	0.86512	Imputed	6460	3.714	2.449	5.631	6.48E−10	9465	1.887	1.322	2.694	0.000473	ncRNA_intronic	LINC01695
21	2,81,65,671	chr21:28165671:C:G	rs2831578	G	0.0066	0.86537	Imputed	6460	3.54	2.359	5.312	1.04E−09	9465	1.789	1.265	2.531	0.001014	ncRNA_intronic	LINC01695
21	2,81,65,241	chr21:28165241:T:A	rs138282731	A	0.00622	0.86591	Imputed	6460	3.714	2.449	5.631	6.48E−10	9465	1.887	1.322	2.694	0.000473	ncRNA_intronic	LINC01695
21	2,81,67,027	chr21:28167027:G:A	rs2831584	A	0.006	0.87016	Imputed	6460	3.653	2.385	5.596	2.61E−09	9465	1.878	1.306	2.699	0.000666	ncRNA_intronic	LINC01695
21	2,81,68,119	chr21:28168119:C:A	rs2831588	A	0.00661	0.8744	Imputed	6460	3.311	2.188	5.01	1.47E−08	9465	1.746	1.235	2.468	0.001598	ncRNA_intronic	LINC01695
21	2,81,70,454	chr21:28170454:C:T	rs2831597	T	0.00518	0.88171	Imputed	6460	4.405	2.798	6.935	1.52E−10	9465	2.218	1.501	3.277	6.37E−05	ncRNA_intronic	LINC01695
21	2,81,71,717	chr21:28171717:A:G	rs57177980	G	0.00563	0.88918	Imputed	6460	3.752	2.382	5.908	1.15E−08	9465	1.922	1.314	2.809	0.000749	ncRNA_intronic	LINC01695
21	2,84,06,331	chr21:28406331:T:C	rs150168760	C	0.0048	0.89787	Imputed	6460	4.157	2.613	6.614	1.80E−09	9465	1.735	1.151	2.614	0.00844	Intergenic	LINC01695;LINC00161
21	2,81,87,913	chr21:28187913:T:C	rs10482989	C	0.00602	0.93268	Imputed	6460	3.599	2.324	5.572	9.50E−09	9465	2.024	1.405	2.916	0.000155	ncRNA_intronic	LINC01695
21	2,81,89,835	chr21:28189835:CT:C		C	0.00604	0.93481	Imputed	6460	3.599	2.324	5.572	9.50E−09	9465	2.024	1.405	2.916	0.000155	ncRNA_intronic	LINC01695
21	2,81,99,184	chr21:28199184:A:T	rs28883424	T	0.00564	0.9351	Imputed	6460	3.761	2.405	5.881	6.35E−09	9465	2.012	1.384	2.925	0.000252	ncRNA_intronic	LINC01695
21	2,81,90,781	chr21:28190781:TAC:T		T	0.00502	0.93737	Imputed	6460	4	2.524	6.338	3.58E−09	9465	1.999	1.354	2.951	0.000494	ncRNA_intronic	LINC01695
21	2,81,99,308	chr21:28199308:A:G	rs8130449	G	0.00605	0.9382	Imputed	6460	3.599	2.324	5.572	9.50E−09	9465	2.024	1.405	2.916	0.000155	ncRNA_intronic	LINC01695
21	2,81,97,976	chr21:28197976:C:T	rs59067393	T	0.00502	0.93881	Imputed	6460	4	2.524	6.338	3.58E−09	9465	1.999	1.354	2.951	0.000494	ncRNA_intronic	LINC01695
21	2,82,03,693	chr21:28203693:G:A	rs78202304	A	0.00578	0.95837	Imputed	6460	3.82	2.454	5.948	2.97E−09	9465	2.141	1.478	3.101	5.69E−05	ncRNA_intronic	LINC01695
21	2,82,25,654	chr21:28225654:C:T	rs73897628	T	0.00536	0.96681	Imputed	6460	3.635	2.309	5.721	2.45E−08	9465	1.739	1.185	2.551	0.004699	ncRNA_intronic	LINC01695
21	2,83,52,351	chr21:28352351:G:T	rs7278151	T	0.00874	0.96719	Imputed	6460	2.909	2.029	4.172	6.37E−09	9465	1.379	1.015	1.873	0.04014	Intergenic	LINC01695;LINC00161
21	2,82,06,978	chr21:28206978:G:A	rs144947925	A	0.00515	0.97068	Imputed	6460	3.938	2.507	6.185	2.66E−09	9465	1.939	1.323	2.842	0.000692	ncRNA_intronic	LINC01695
21	2,82,06,642	chr21:28206642:G:C	rs16997642	C	0.00558	0.97287	Imputed	6460	3.866	2.484	6.019	2.12E−09	9465	2.031	1.4	2.946	0.000189	ncRNA_intronic	LINC01695
21	2,82,22,390	chr21:28222390:T:C	rs2831662	C	0.00587	0.97928	Imputed	6460	3.606	2.318	5.611	1.30E−08	9465	1.725	1.193	2.496	0.003782	ncRNA_intronic	LINC01695
21	2,83,36,083	chr21:28336083:T:C	rs73897689	C	0.00493	0.98436	Imputed	6460	3.822	2.406	6.072	1.38E−08	9465	1.792	1.208	2.658	0.003747	Intergenic	LINC01695;LINC00161
21	2,83,37,534	chr21:28337534:T:C	rs73897690	C	0.00489	0.98684	Imputed	6460	3.822	2.406	6.072	1.38E−08	9465	1.807	1.217	2.683	0.003374	Intergenic	LINC01695;LINC00161
21	3,45,50,989	chr21:34550989:C:T	rs140276394	T	0.0032	0.50721	Imputed	6460	6.028	3.245	11.2	1.31E−08	9465	1.888	1.025	3.478	0.04147	Intronic	RCAN1

### Replication of the PRS model and additional novel loci

Consequently, we switched the two cohorts, i.e. using the second cohort for the statistics of PRS modelling, then we tested the PRS models in the first cohort. The AUCs of different cutoffs of T1D association *P* values for selection of SNP sets are shown in Table 1b. The best AUC (0.8654) is seen at the cutoff of *P* value ≤ 1E−05, which repeated the PRS model in the above step. Based on the SNP markers with T1D association *P* value ≤ 1E−05, a PRS score was acquired for each individual in the independent test cohort. By the maximum MCC (Supplementary Table 3), a PRS cutoff of 7.18E−04 has the maximum MCC (0.6294). A PRS ≤ 7.18E−04 was defined as low risk, and a PRS > 7.18E−04 was defined as high risk. With this threshold, the sensitivity (True positive rate, TPR) for T1D prediction is 66.0%, and the specificity (True negative rate, TFR) for T1D prediction is 93.6%. By PRS ≤ 7.18E−04, 907 (27.5%, including 433 males, 472 females, and 2 cases with undetermined sex) out of 3302 T1D cases had low PRS; and 5567 (90.1%, including 2997 males, 2558 females, and 12 cases with undetermined sex) out of 6181 controls had low PRS.

**Figure 3:**
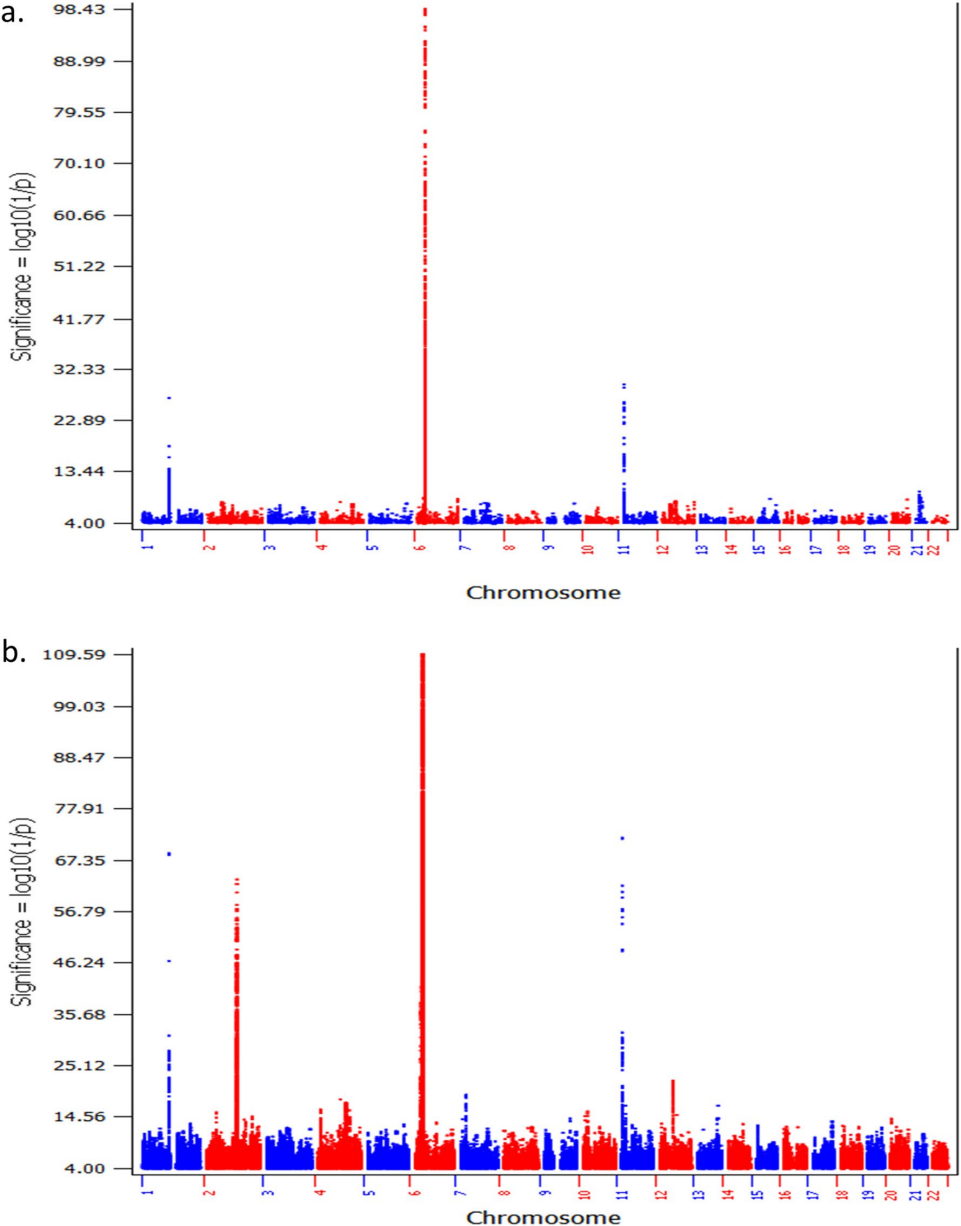
The Manhattan plots of cohort A. (**a**) The plot of the GWAS of T1D patients with low T1D PRS compared to controls with low T1D PRS (907 cases vs. 5567 controls); (**b**) the plot of the GWAS of all T1D patients compared to all controls (3302 cases vs. 6181 controls).

As expected from the above results, in the switched cohort, the GWAS of T1D patients with low T1D PRS compared to controls with low T1D PRS identified a large number of SNPs associated with T1D with genome-wide significance (*P* ≤ 5.0 × E−08) as well (Supplementary Table  4, Fig. 3). Among these loci, 4 loci have been established of T1D association by previous studies, including *HLA*, *INS*, *PTPN22*, *IKZF4/RPS26/ERBB3*, and the locus (Table 2b). Consistent to the first GWAS results listed above, by looking at the established leading T1D signal of each locus, the frequencies of the predisposing alleles of *HLA, PTPN22* and *IKZF4* were lower in the low T1D PRS cohort, while the protective allele of *INS* were higher in the low T1D PRS cohort. The effect size of the leading *HLA* SNP was significantly smaller in the low PRS cases (*P* = 5.49E−11). Besides these established T1D loci, 18 loci associated with low PRS T1D were identified in this cohort (Table 3b). LocusZoom plots for genetic association signals within each locus are shown in Supplementary Figures 8–25. Among the 18 loci, 16 loci are novel, while the Notch ligand Delta-like 1 (*DLL1*) locus, with the gene function essential for pancreatic islet homeostasis^23^, have been identified by our gene-based association study on low PRS T1D^24^. The other locus, containing the UDP-GlcNAc:betaGal beta-1,3-N-acetylglucosaminyltransferase 2 gene (*B3GNT2*) and transmembrane protein 17 gene (*TMEM17*), is ~ 200 kb from the Eps15 homology domain binding protein 1 locus (*EHBP1*) that has been identified of genome-wide significance in our study on low T1D GRS patients^21^.

## Discussion

Altogether, rare variants (MAF < 5%) from 22 novel loci were identified in the low PRS T1D cases with genome-wide significance (*P* < 5.00E−08), in addition to the 4 established T1D loci, 2 reported loci in the low GRS patients, and 1 locus by our gene-based study. The genome-wide significant association signals of these loci are only seen in low PRS T1D cases, but not in the T1D cases overall, thus were missed previously due to rare allele frequencies and diluted genetic effects in the general T1D cohort. A number of genetic associations with body mass index (BMI), obesity, and autoimmunity, have been reported in the flanking regions of 300 kb on each side of these new loci according to the GWAS Catalog (https://www.ebi.ac.uk/gwas/, Supplementary materials for review). Further discussion on these novel loci is focused on 13 loci with high imputation quality (i.e. Quality Score r^2^ > 0.9). Among these loci, 9 loci are related to obesity traits (T1bD mechanism), 2 loci are related to glucose homeostasis (T1bD mechanism), and 2 loci are related to autoimmunity (T1aD mechanism).

### Obesity-related/ T1bD-related loci

#### *FAM49A/RAD51AP2* tagged by rs56806432

Two coding genes in this locus are the CYFIP related Rac1 interactor A gene (*CYRIA*) and the RAD51 associated protein 2 gene (*RAD51AP2*). *CYRIA* is highly expressed in brain and thyroid, while *RAD51AP2* has restricted expression toward testis^25^. Previous GWAS has identified association of this locus with subcutaneous adipose tissue^26^.

#### *NFIB* tagged by rs10961435

The nuclear factor I B gene (*NFIB*) encodes a transcription factor in the FOXA1 transcription factor network. NFIB has been shown to play critical roles in lung and brain development. A previous study has shown that NFIB can bind with FoxA1 and modulate the transcriptional activity of FoxA1^27^, while the later has been suggested to play a role in pancreatic and ß-cell function and non-autoimmune diabetes as discussed above. The nuclear factor I B gene (NFIB) has ubiquitous expression in fat, brain, and other tissues^25^. This locus has been reported of association with BMI by several GWA studies^28–30^.

#### *LINC00841/C10orf142* tagged by rs746298

The two genes at this locus, *LINC00841/C10orf142,* encode two long intergenic non-protein coding RNAs (lincRNA). While the function of these two genes remain unknown, this locus has been reported of association with obesity-related traits^31^.

#### *FAM136A/TGFA* tagged by rs77418738

The family with sequence similarity 136 member A gene (*FAM136A*) encodes a mitochondrially localized protein^32^. The transforming growth factor alpha gene (TGFA) mediates cell–cell adhesion and activates cell proliferation, differentiation and development^33^. This region has been reported of association with obesity-related traits^31^.

#### *CALN1* tagged by rs118182411

The calneuron 1 gene (*CALN1*), encoding a protein with high similarity to the calcium-binding proteins of calmodulin, is highly expressed in brain and adrenal^25^. This genetic region has established association with BMI by previous studies^29,30^.

#### *EPHB4* tagged by rs3890144

The EPH receptor B4 gene (*EPHB4*) has ubiquitous expression in multiple tissues, and is involved in numerous developmental processes^34^. *EPHB4* plays critical roles in vascular development^35^ and lymphatic valve development^36^. Previous GWAS has identified association of this locus with BMI^37^ and waist circumference adjusted for BMI^30^.

#### *TXN/TXNDC8* tagged by rs10816957

The thioredoxin gene (TXN) has ubiquitous expression in multiple tissues, while the thioredoxin domain containing 8 gene (TXNDC8) has restricted expression toward testis^25^. Thioredoxin plays a protective role against oxidative stresses^38^. Thioredoxin interacting protein (TXNIP) has been implicated in β cells death in diabetes and is a novel potential therapeutic target of diabetes^39^. Previous GWAS has identified association of this locus with BMI^40^ and waist-to-hip ratio adjusted for BMI^30^.

#### *SYT10/ALG10* tagged by rs4142676

The synaptotagmin 10 gene (*SYT10*) encodes a membrane protein of secretory vesicles expressed in pancreas, lung and kidney^41^. The ALG10 alpha-1,2-glucosyltransferase gene (*ALG10*) encodes a membrane-associated protein that adds the third glucose residue to the lipid-linked oligosaccharide precursor for N-glycosylation in endoplasmic reticulum (ER)^42^. As discussed above in the *ZNF804B* locus, N-glycosylation of IgG, cytokines and proteases is also a regulatory mechanism in inflammation and autoimmunity^43,44^ associated with different autoimmune diseases. Several previous GWASs have identified association of this locus with waist-to-hip ratio and waist-to-hip ratio adjusted for BMI^30,45^.

#### *CHFR/LOC101928530/ZNF605* tagged by rs12230138

The checkpoint with forkhead and ring finger domains gene (*CHFR*) encodes an E3 ubiquitin-protein ligase and is involved in the DNA damage response and checkpoint regulation^46^. The structure and function of the gene *LOC101928530* is still uncharacterized. The function of the zinc finger protein 605 gene (*ZNF605*) may be related to Herpes Simplex Virus 1 infection (https://pathcards.genecards.org/card/herpes_simplex_virus_1_infection). This region has been reported of association with BMI by previous study^28^.

### Genetic loci related to glucose homeostasis (T1bD-related)

#### *LOC730100* tagged by rs28957087

*LOC730100* encodes a long non-coding RNA (ncRNA), a competing endogenous RNA for human microRNA 760 (miR-760)^47^. The latter inhibits the expression of the Forkhead Box A1 gene (*FOXA1*). As a hepatocyte nuclear factor, *FOXA1*, also known as *HNF3A* or *TCF3A,* regulates tissue-specific gene expression in liver and many other tissues^48^. FoxA1 is essential for normal pancreatic and ß-cell function and a negative regulator of the hepatocyte nuclear factor-1 (HNF1) homeobox A gene (*HNF1A*) and the hepatocyte nuclear factor 4, alpha gene (*HNF4A*)^49,50^. *HNF1A* and *HNF4A* are established genes causing maturity-onset diabetes of the young (MODY). The *FOXA1* mutation Ser448Asn has been suggested of association with impaired glucose homeostasis^50^.

#### *LINC01695/LINC00161* tagged by rs2831597

Function of the long intergenic non-protein coding RNA 1695 gene (*LINC01695*) is still uncharacterized. The long intergenic non-protein coding RNA 161 gene (*LINC00161*) encodes a functional RNA that regulates Mitogen-activated protein kinase 1 (MAPK1) expression^51^. The MAPK1/STAT3 pathway has been proposed as a novel diabetes target for its critical role in glucose homeostasis^52^.

### Autoimmune-related loci

In addition to the above *ALG10* locus associated with both autoimmune diseases and obesity-related traits, two other loci were identified in the low PRS T1D cases. The rare variants in these loci may represent rare forms of autoimmune diabetes with low T1D PRS^53^.

#### *LINC02432/IL15* tagged by rs9790756

The long intergenic non-protein coding RNA 2432 gene (*LINC02432*) has higher expression in kidney and pancreas^25^. Interleukin 15 (IL-15) encoded by the gene *IL15* is essential for regulating activation and proliferation of T and natural killer cells, and supporting lymphoid homeostasis^54^. IL-15 and interleukin 2 (IL-2) share many biological activities and receptor components with IL-2^55^. IL-2 is a powerful growth factor for both T and B lymphocytes^56^. Both IL2 and the α chain of the IL2 receptor complex gene (*IL2RA*) has been established of genetic association with T1D by previous studies^57–59^.

#### *ZNF804B* tagged by rs76060515

The zinc finger protein 804B gene (*ZNF804B*) has been reported of association with N-linked glycosylation of human immunoglobulin G (IgG), which modulates its binding to Fc receptors^43^. N-glycosylation of cytokines and proteases is also a regulatory mechanism in inflammation and autoimmunity^44^. Changes in N-glycosylation have been associated with different autoimmune diseases, including rheumatoid arthritis^60^, type 1 diabetes^61^, Crohn's disease^62^.

In summary, in the genetic regions containing the 13 novel loci with high imputation quality disclosed by this study, 9 of these regions have been reported of association with obesity-related traits, BMI, or waist circumference. The correlation with obesity related traits or impaired glucose homeostasis is in keeping with non-autoimmune roles in the diabetes patients with low T1D PRS. Interestingly, the genes *ZNF804B* and *ALG10* related to N-linked glycosylation are highlighted in this study, which may suggest the role of N-glycosylation in impaired glucose homeostasis and pediatric diabetes, while N-glycosylation is commonly altered in diabetes^63^. In addition, 3 loci encoding long intergenic non-protein coding RNAs (lncRNA) identified in this study emphasize the importance of lncRNAs in these diabetes patients. However, we admit that this study has limitations related to the bottleneck of sample size and data resources. The novel loci reported in this study still need replication in independent samples. In addition, the functional mechanisms of these genetic loci in diabetes warrant experimental investigation. Due to the lack of data of T1D autoantibodies in the subjects, the mixture of rare forms of autoimmune diabetes (e.g. monogenic autoimmunity^53^) in addition to non-autoimmune diabetes may exist as suggested by the identification of rare variants in autoimmune-related genes.

## Supplementary Information

Supplementary Figures.

Supplementary Table 1.

Supplementary Table 2.

Supplementary Table 3.

Supplementary Table 4.

Supplementary Information 1.

## Data Availability

All data generated or analyzed during this study are included in this published article.
