# Supplementary material for: Insights into non-autoimmune type 1 diabetes with 13 novel loci in low polygenic risk score patients
Source: Sci Rep. 2021 Aug 6;11:16013. doi: 10.1038/s41598-021-94994-9 (PMC8346538; doi:10.1038/s41598-021-94994-9)
Supplement: Supplementary file 1 — Supplementary Figures. [file 41598_2021_94994_MOESM1_ESM.pdf]

Insights into Non-autoimmune Type 1 Diabetes with 13 Novel Loci in Low Polygenic Risk Score Patients

Jingchun Qu, Hui-Qi Qu, Jonathan P Bradfield, Joseph T Glessner, Xiao Chang, Lifeng Tian, Michael March, John J Connolly, Jeffrey D Roizen, Patrick MA Sleiman, Hakon Hakonarson

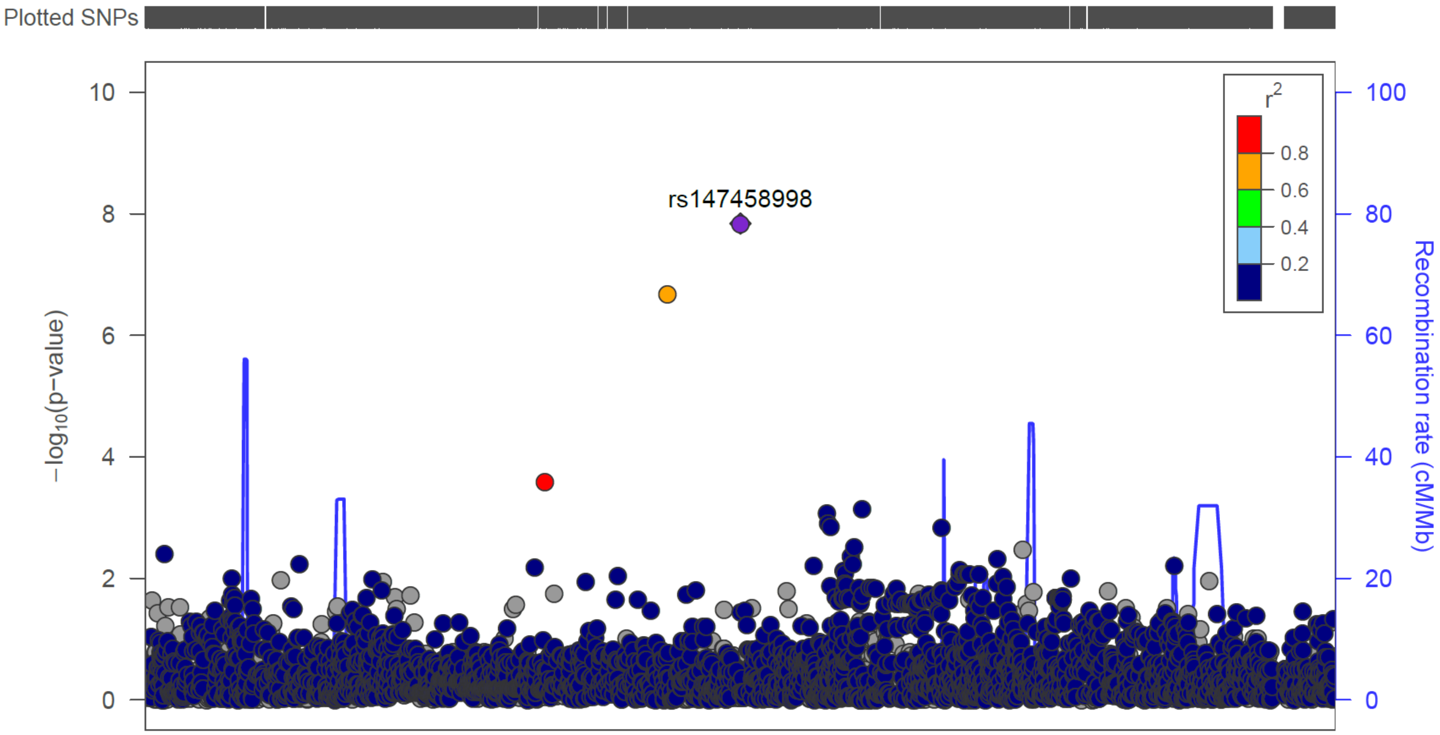

a.

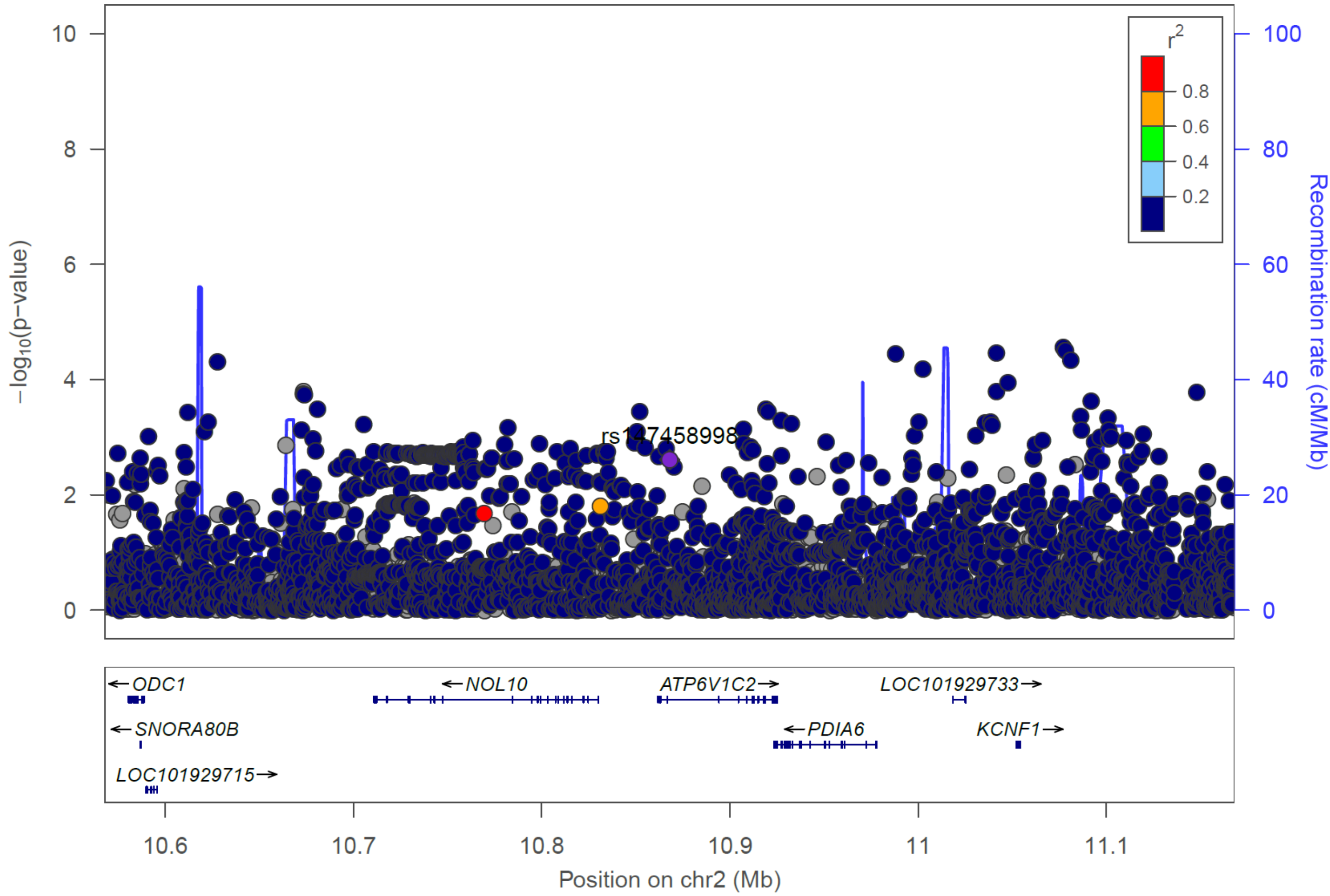

b.

Supplementary Figure 1. The LocusZoom plots for the *ATP6V1C2* locus (rs147458998). (a) The plot of the association tests of T1D patients with low T1D PRS compared to controls with low T1D PRS; (b) The plot of the association tests of all T1D patients compared to all controls.

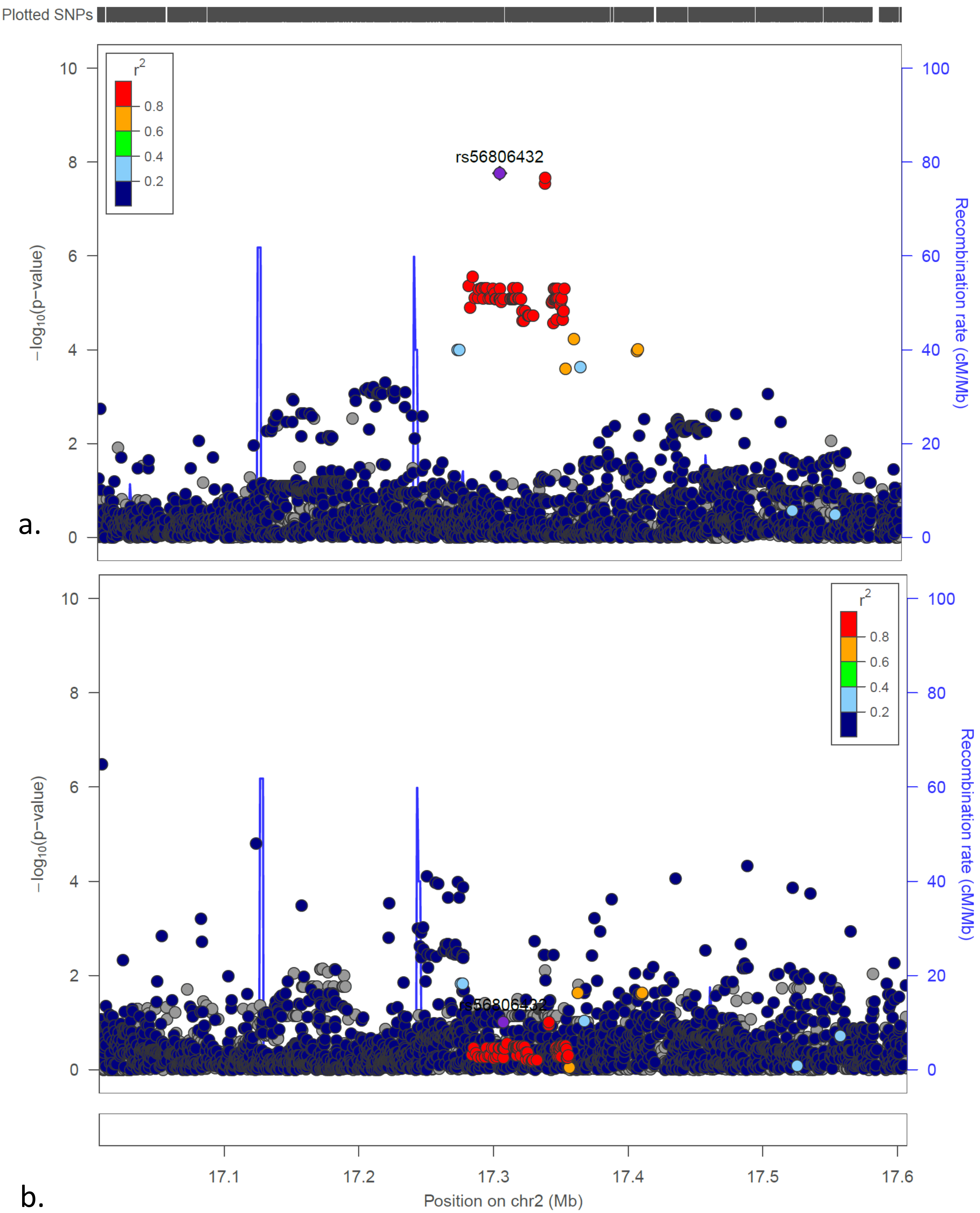

Supplementary Figure 2. The LocusZoom plots for the *FAM49A/RAD51AP2* locus (rs56806432). (a) The plot of the association tests of T1D patients with low T1D PRS compared to controls with low T1D PRS; (b) The plot of the association tests of all T1D patients compared to all controls.

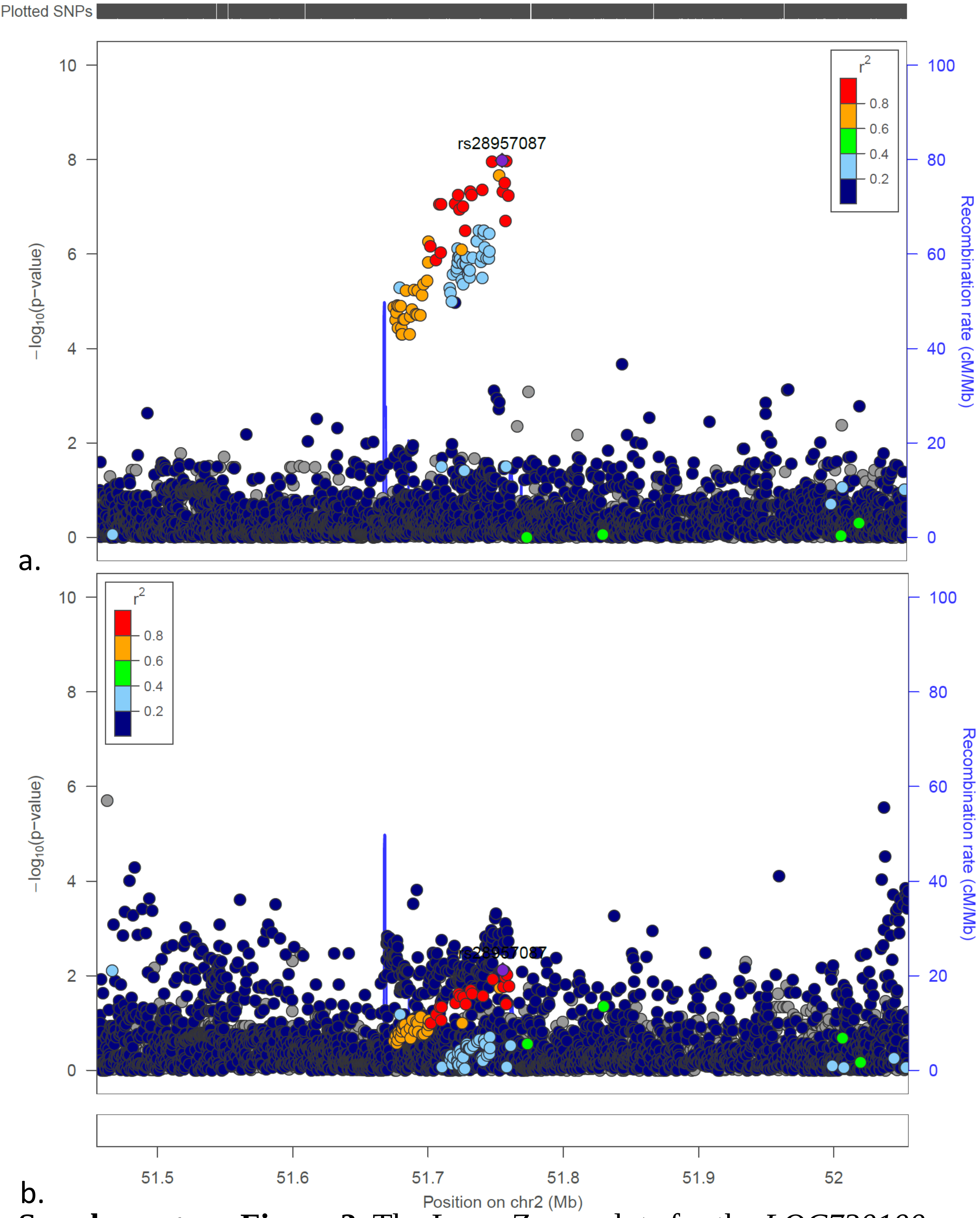

Supplementary Figure 3. The LocusZoom plots for the *LOC730100* locus (rs28957087). (a) The plot of the association tests of T1D patients with low T1D PRS compared to controls with low T1D PRS; (b) The plot of the association tests of all T1D patients compared to all controls.

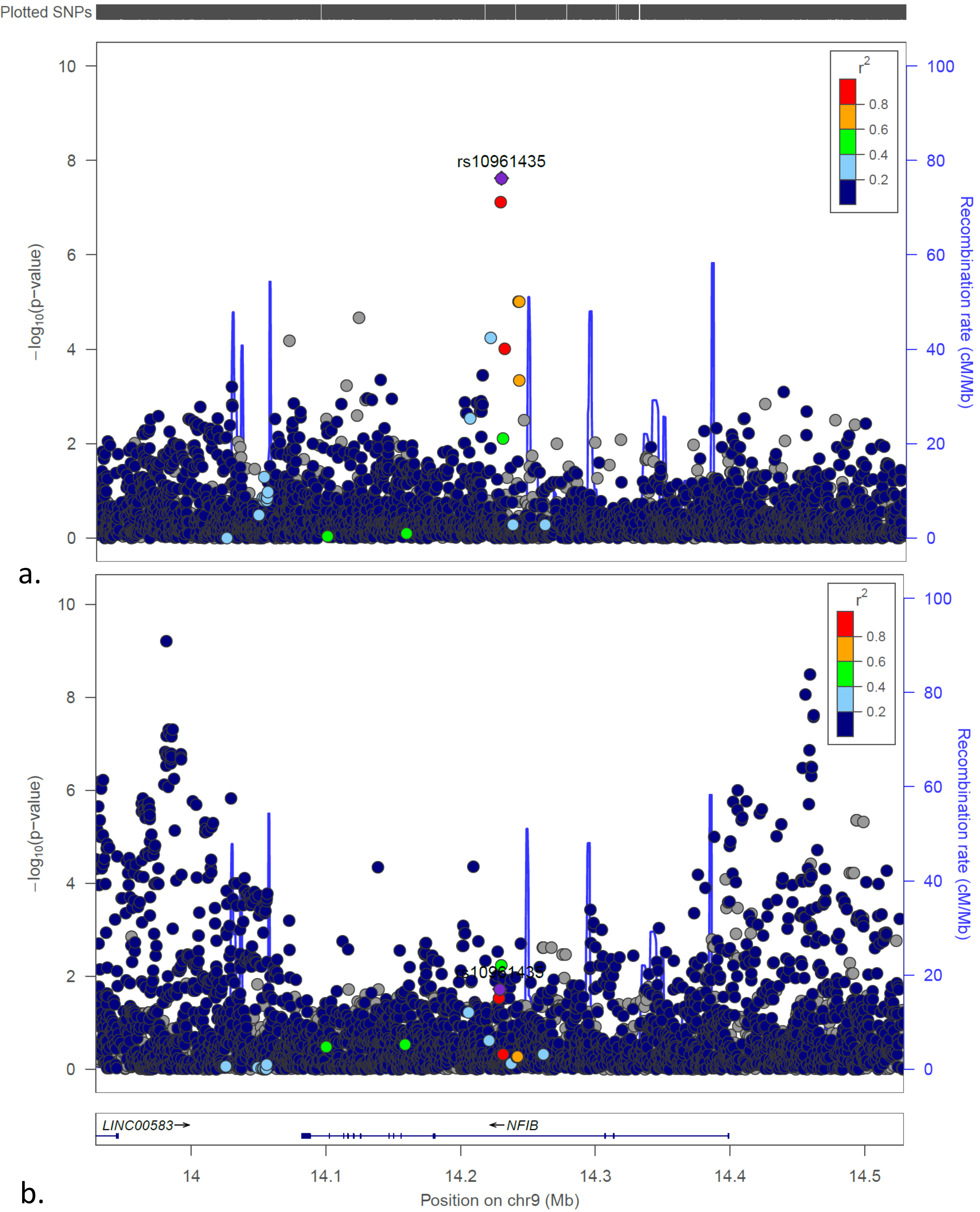

Supplementary Figure 4. The LocusZoom plots for the *NFIB* locus (rs10961435). (a) The plot of the association tests of T1D patients with low T1D PRS compared to controls with low T1D PRS; (b) The plot of the association tests of all T1D patients compared to all controls.

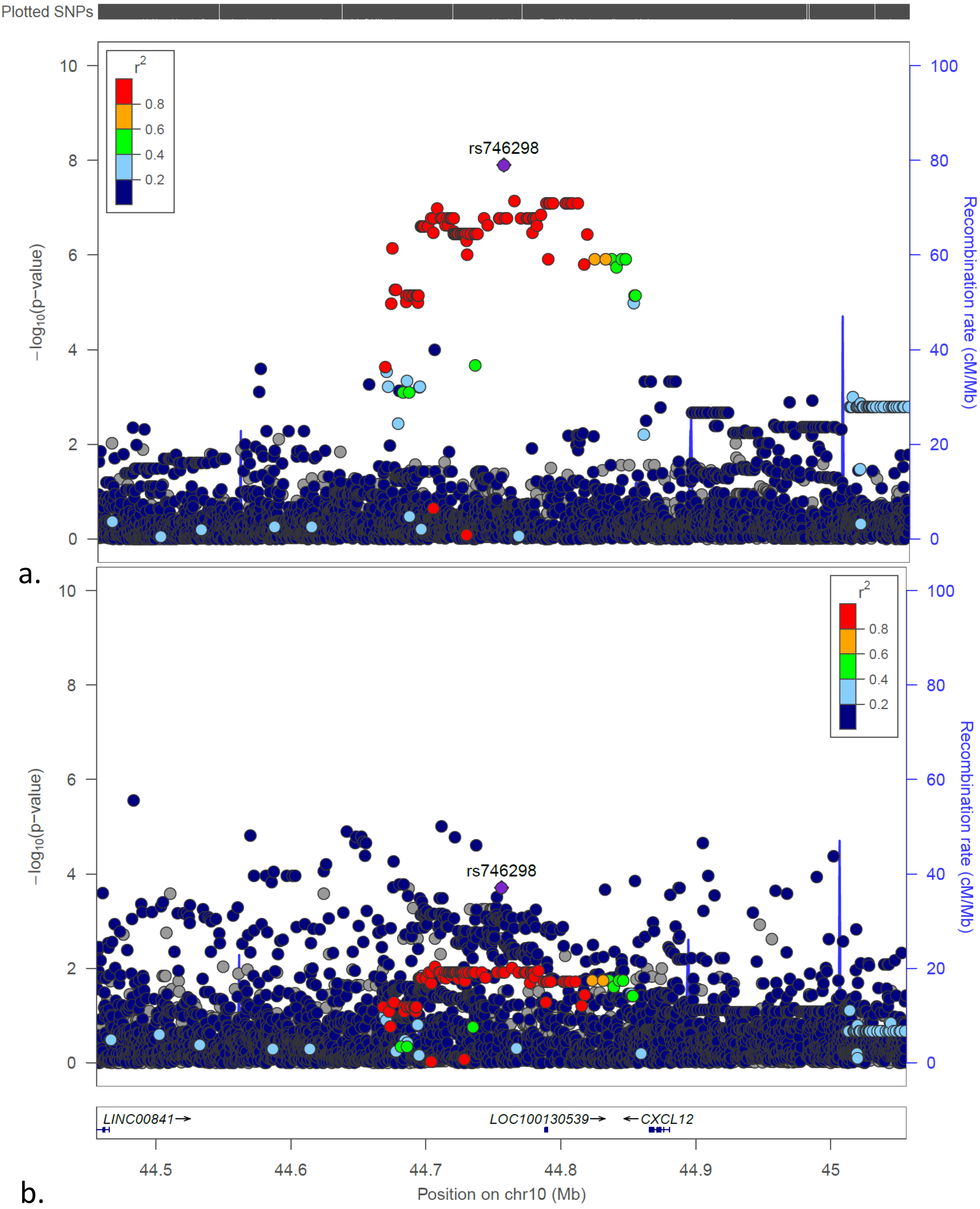

Supplementary Figure 5. The LocusZoom plots for the *LINC00841/C10orf142* locus (rs746298). (a) The plot of the association tests of T1D patients with low T1D PRS compared to controls with low T1D PRS; (b) The plot of the association tests of all T1D patients compared to all controls.

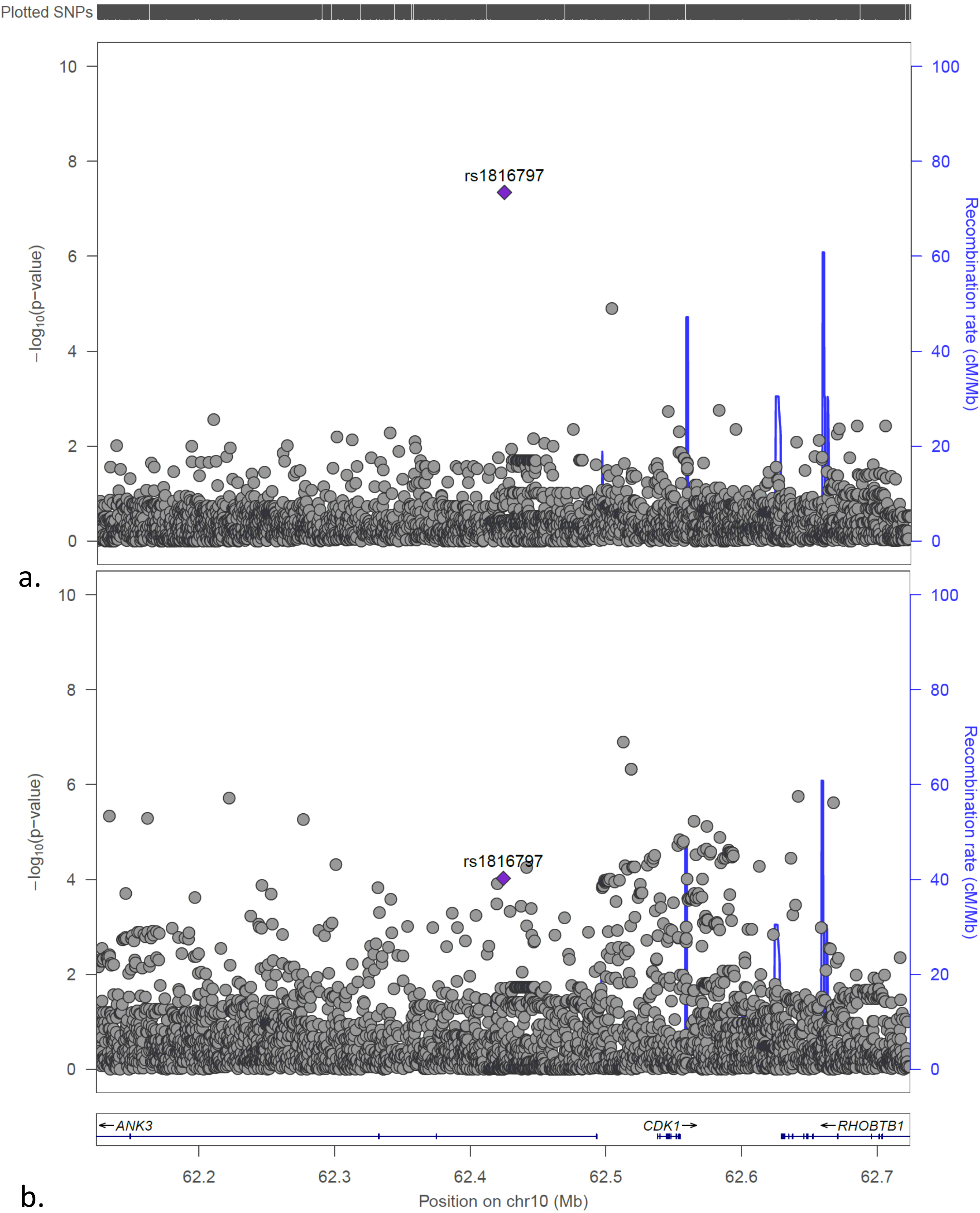

Supplementary Figure 6. The LocusZoom plots for the *ANK3* locus (rs1816797). (a) The plot of the association tests of T1D patients with low T1D PRS compared to controls with low T1D PRS; (b) The plot of the association tests of all T1D patients compared to all controls.

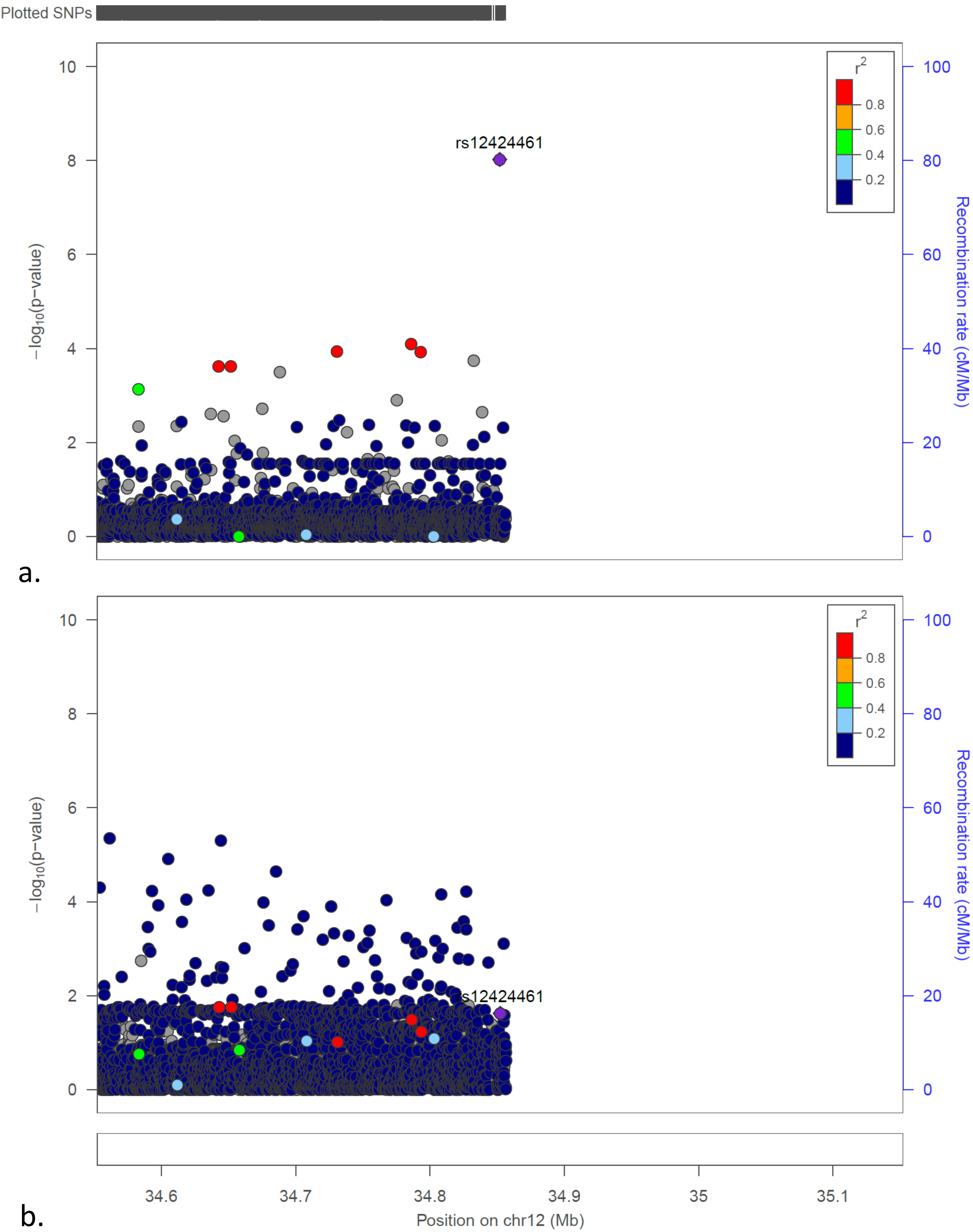

Supplementary Figure 7. The LocusZoom plots for the *ALG10* locus (rs12424461). (a) The plot of the association tests of T1D patients with low T1D PRS compared to controls with low T1D PRS; (b) The plot of the association tests of all T1D patients compared to all controls.

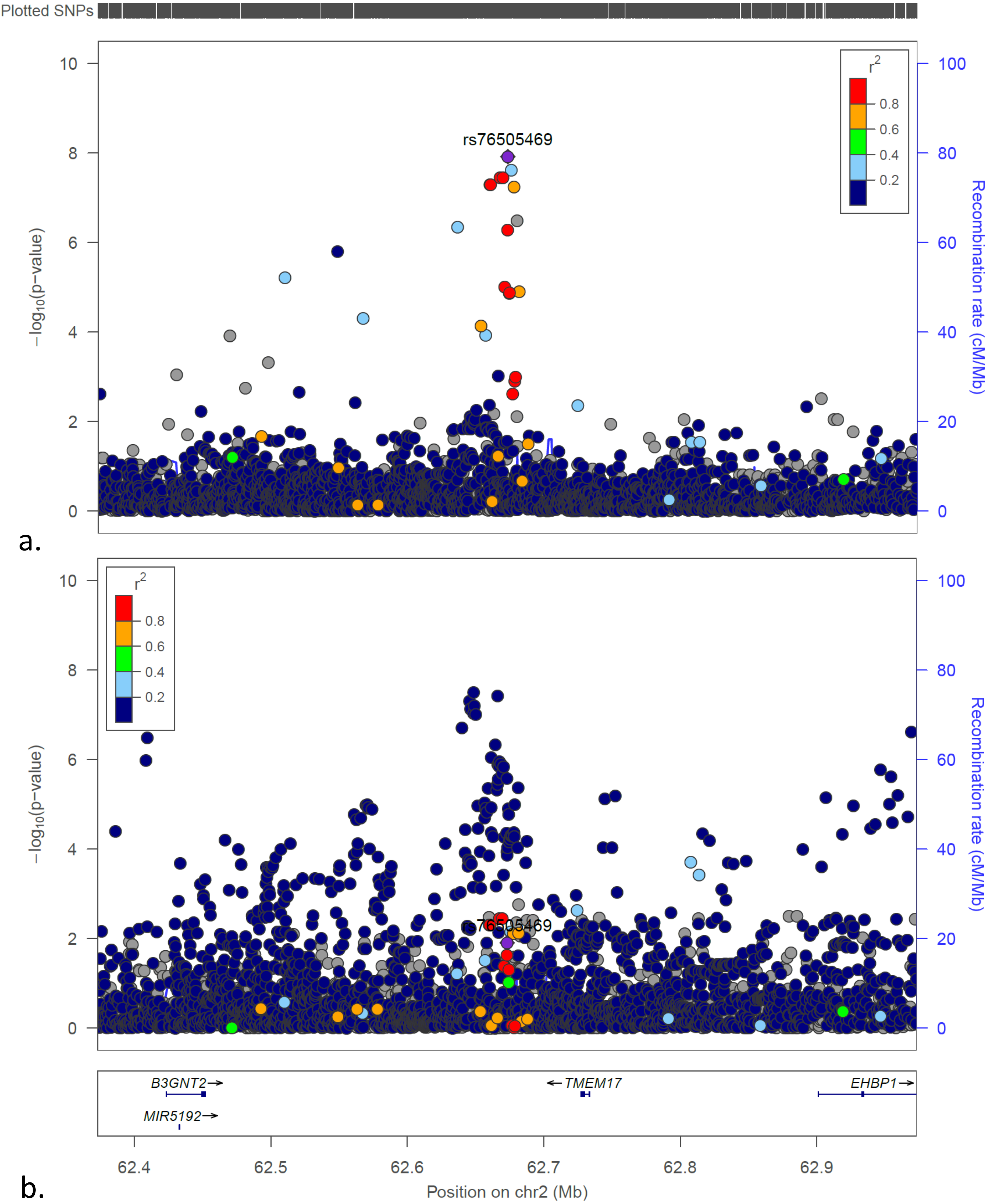

Supplementary Figure 8. The LocusZoom plots for the *B3GNT2/TMEM17* locus (rs76505469). (a) The plot of the association tests of T1D patients with low T1D PRS compared to controls with low T1D PRS; (b) The plot of the association tests of all T1D patients compared to all controls.

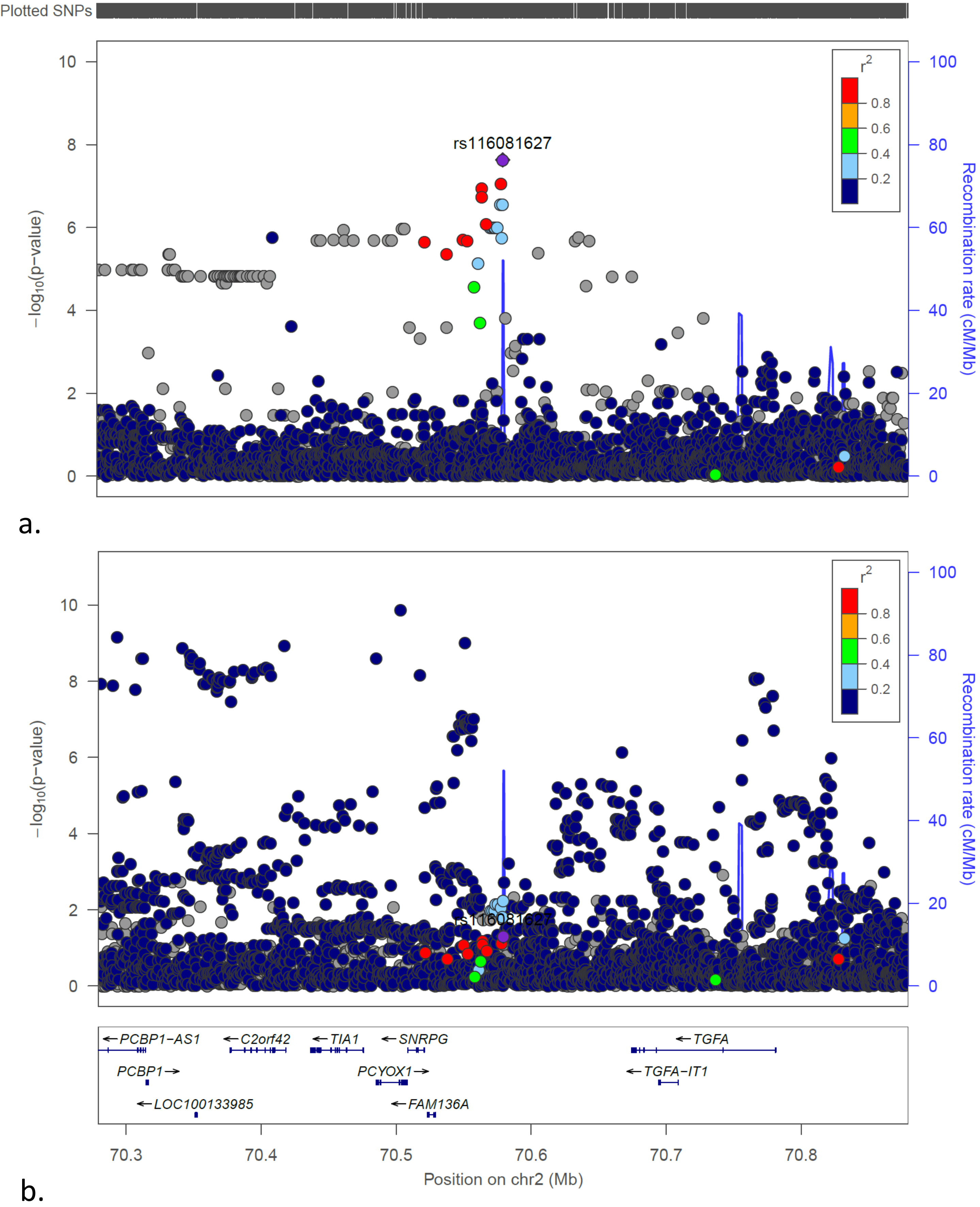

Supplementary Figure 9. The LocusZoom plots for the *FAM136A/TGFA* locus (rs116081627). (a) The plot of the association tests of T1D patients with low T1D PRS compared to controls with low T1D PRS; (b) The plot of the association tests of all T1D patients compared to all controls.

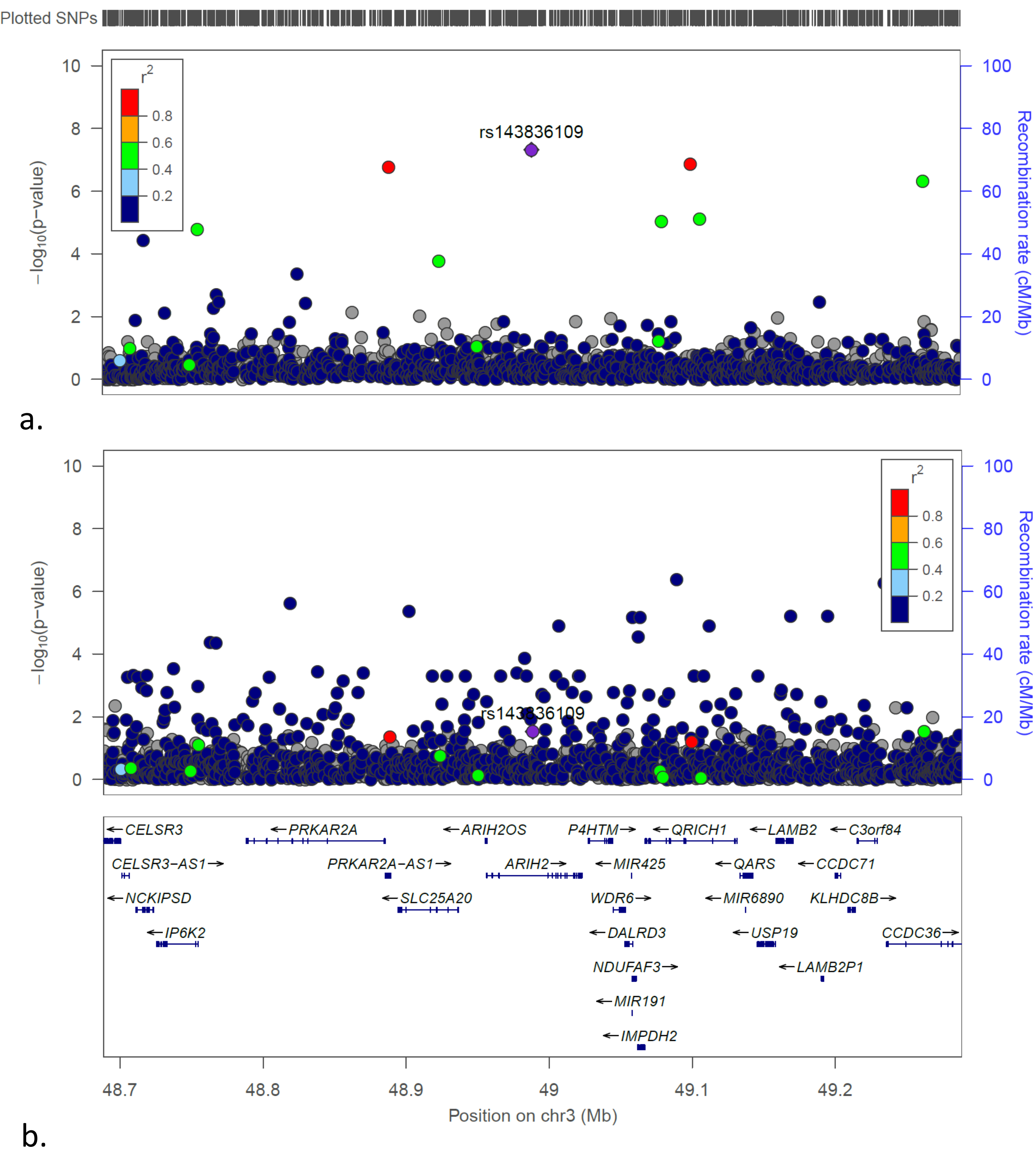

Supplementary Figure 10. The LocusZoom plots for the *ARIH2* locus (rs143836109). (a) The plot of the association tests of T1D patients with low T1D PRS compared to controls with low T1D PRS; (b) The plot of the association tests of all T1D patients compared to all controls.

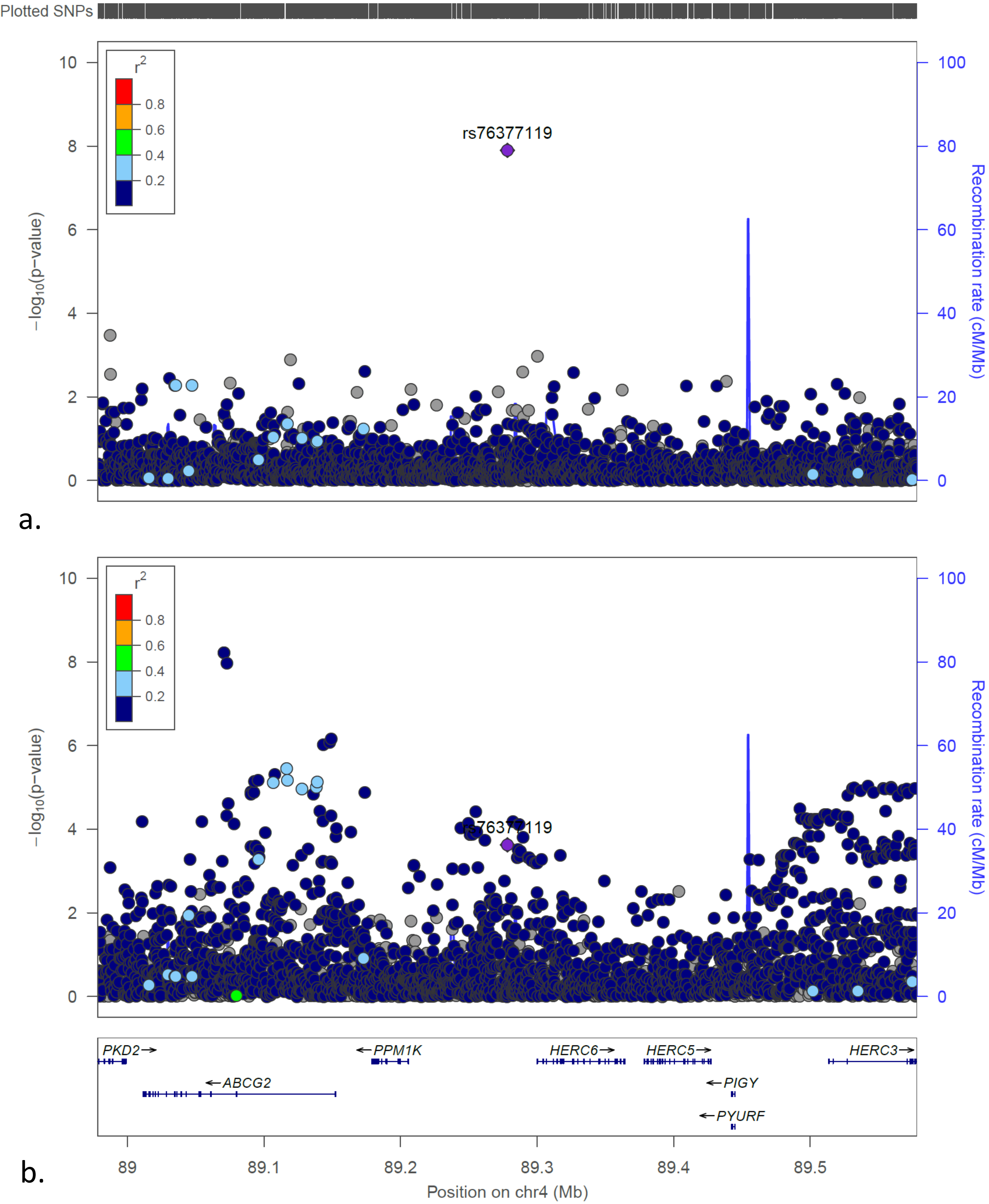

Supplementary Figure 11. The LocusZoom plots for the *LOC105369192/HERC6* locus (rs76377119). (a) The plot of the association tests of T1D patients with low T1D PRS compared to controls with low T1D PRS; (b) The plot of the association tests of all T1D patients compared to all controls.

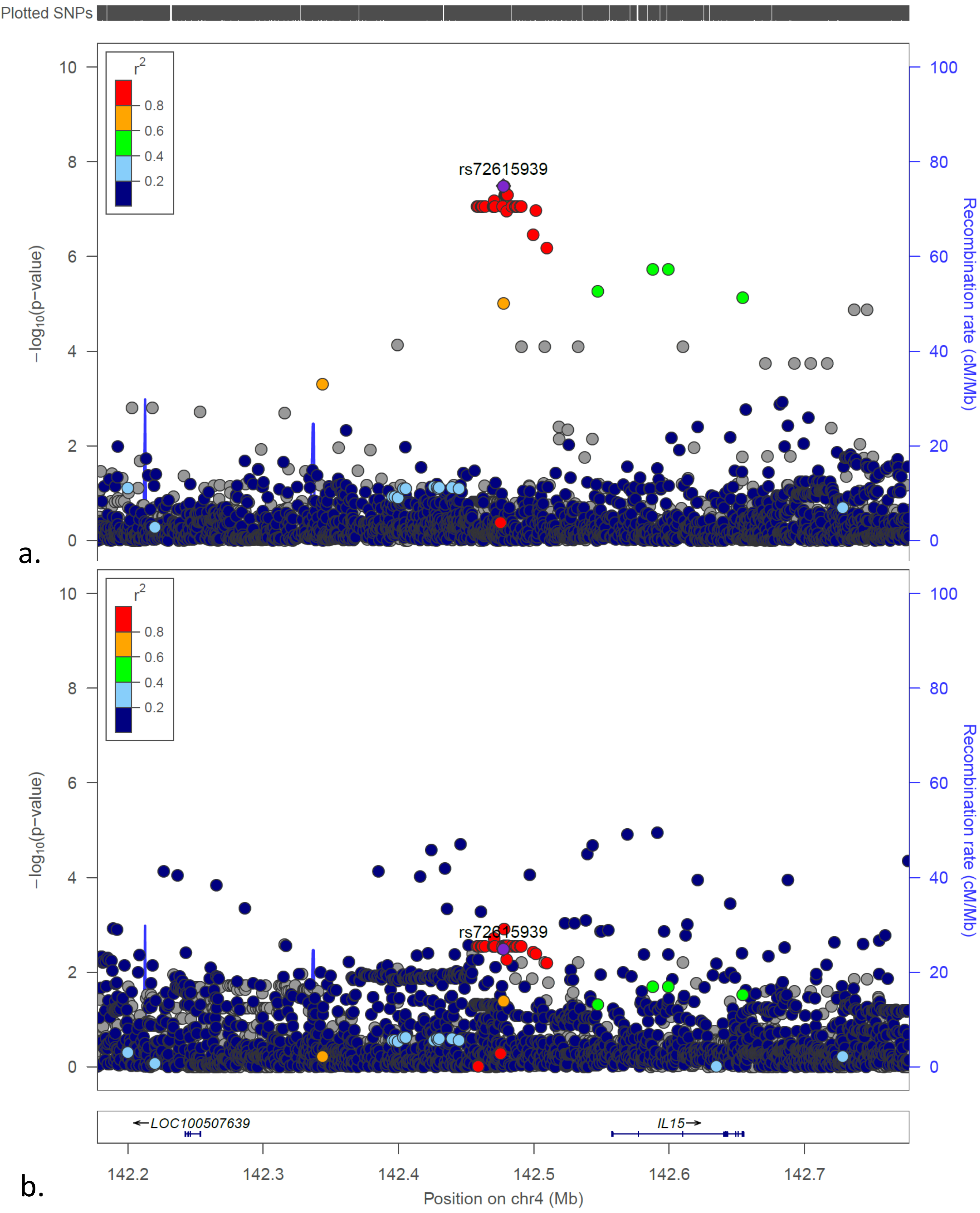

Supplementary Figure 12. The LocusZoom plots for the *LINC02432/IL15* locus (rs72615939). (a) The plot of the association tests of T1D patients with low T1D PRS compared to controls with low T1D PRS; (b) The plot of the association tests of all T1D patients compared to all controls.

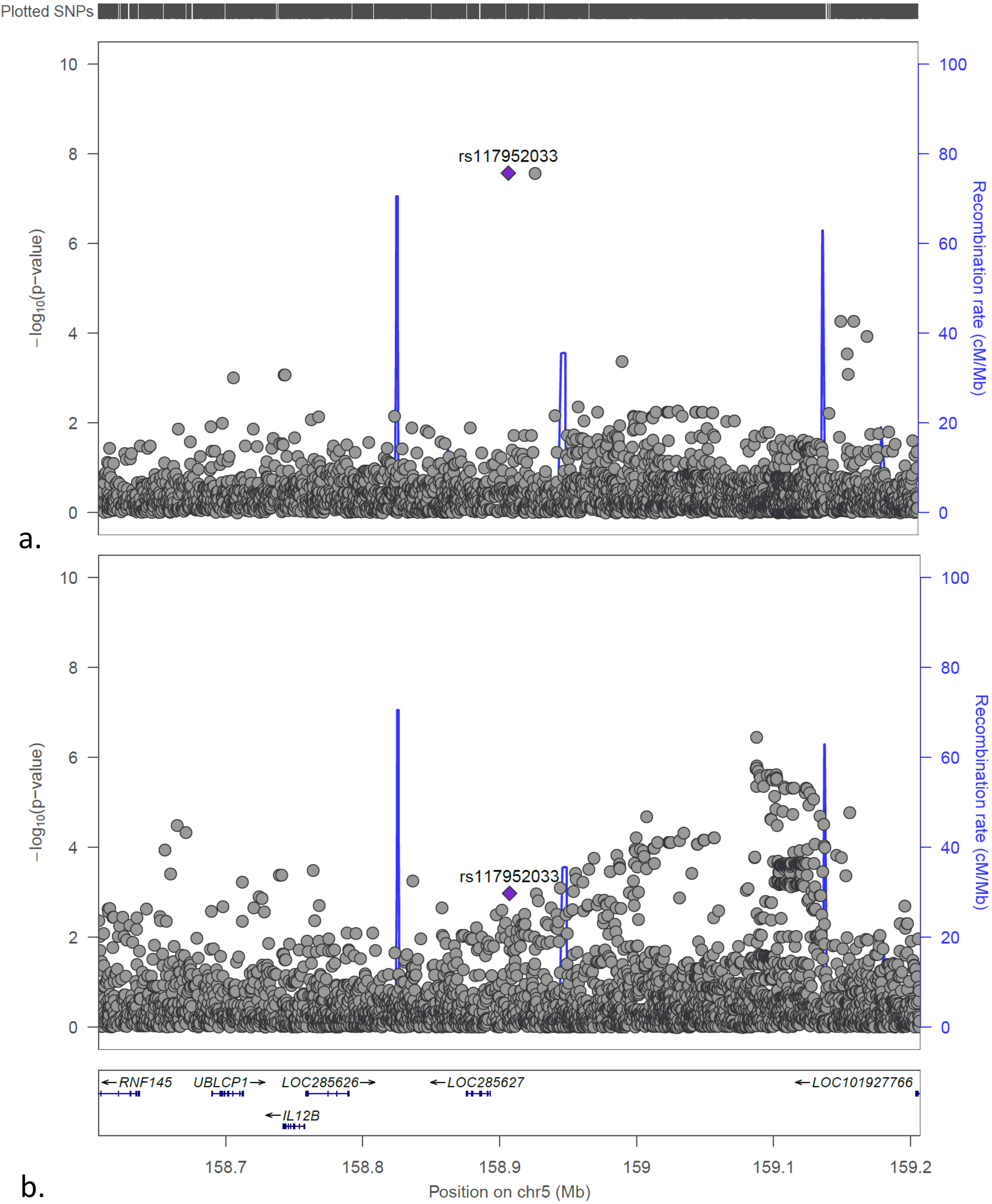

Supplementary Figure 13. The LocusZoom plots for the *LINC01845/LINC01847* locus (rs117952033). (a) The plot of the association tests of T1D patients with low T1D PRS compared to controls with low T1D PRS; (b) The plot of the association tests of all T1D patients compared to all controls.

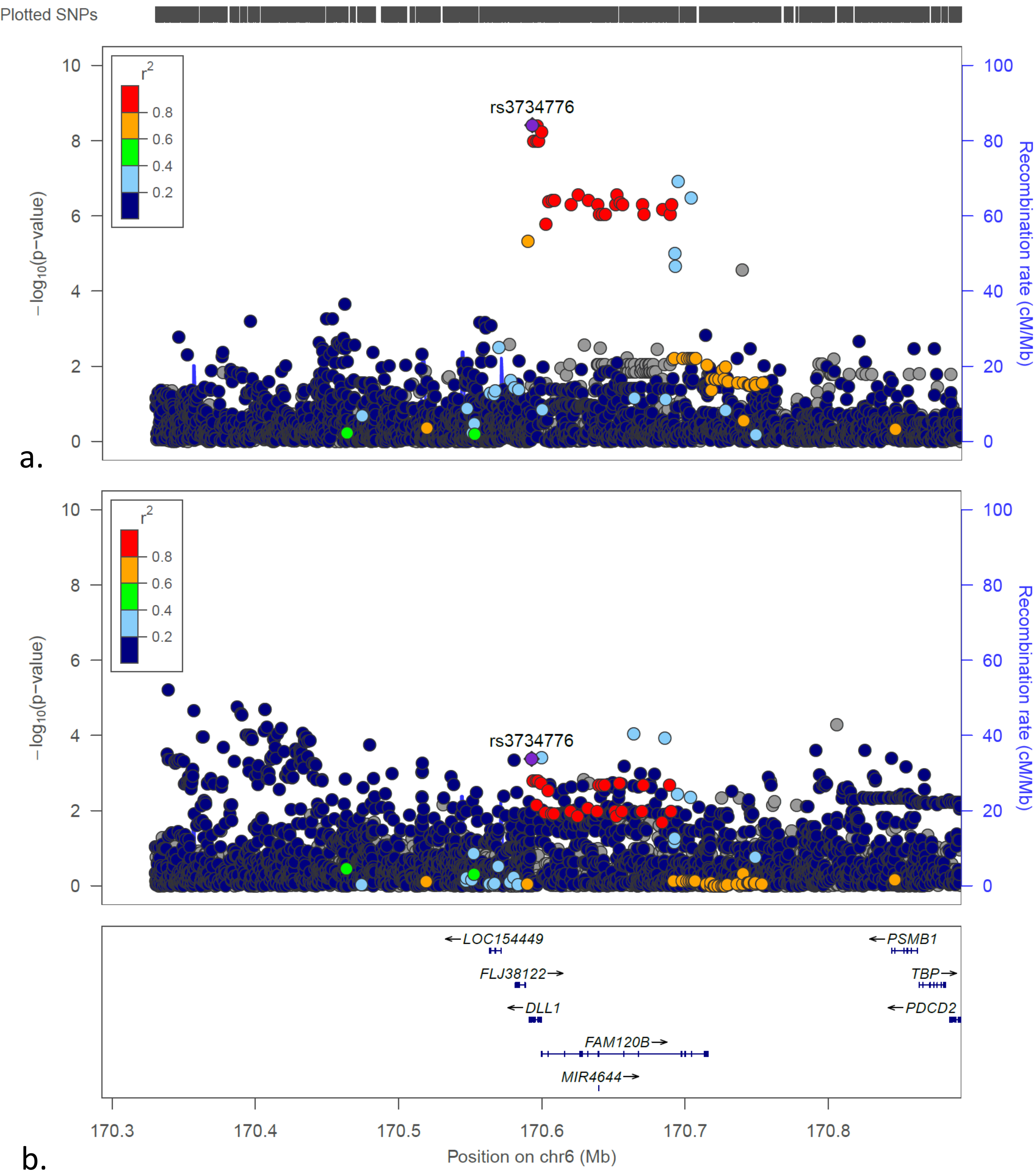

Supplementary Figure 14. The LocusZoom plots for the *DLL1* locus (rs3734776). (a) The plot of the association tests of T1D patients with low T1D PRS compared to controls with low T1D PRS; (b) The plot of the association tests of all T1D patients compared to all controls.

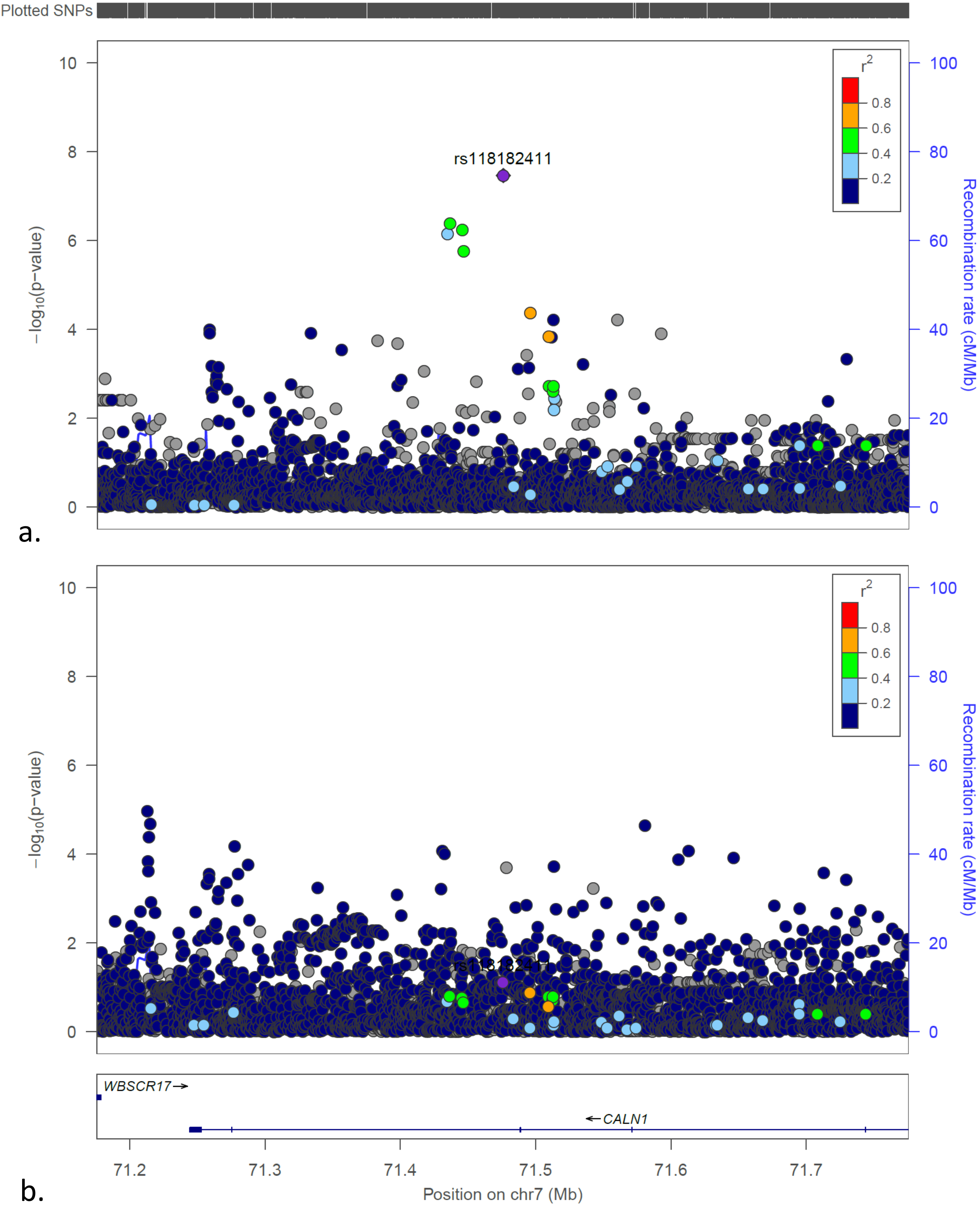

Supplementary Figure 15. The LocusZoom plots for the *CALN1* locus (rs118182411). (a) The plot of the association tests of T1D patients with low T1D PRS compared to controls with low T1D PRS; (b) The plot of the association tests of all T1D patients compared to all controls.

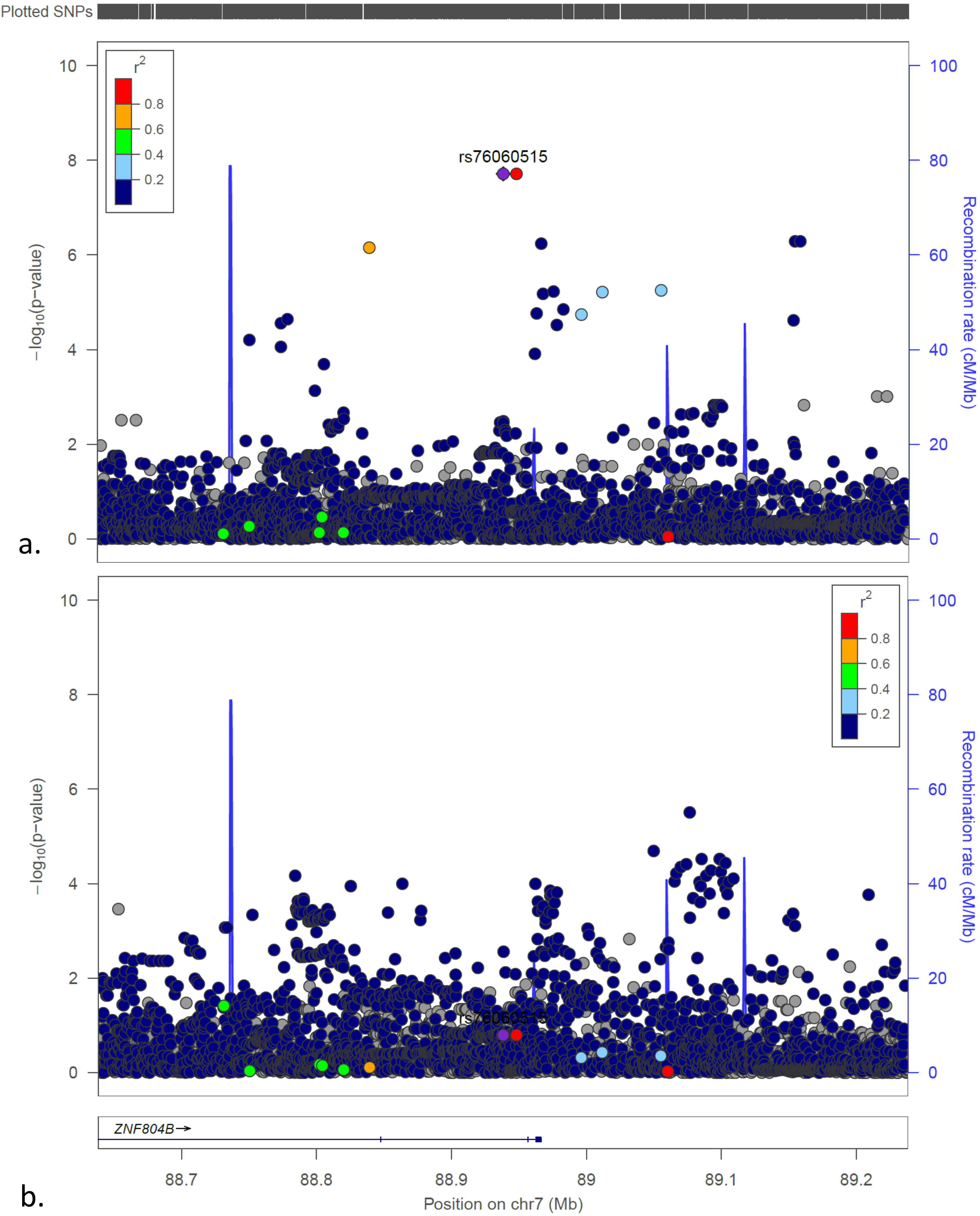

Supplementary Figure 16. The LocusZoom plots for the *ZNF804B* locus (rs76060515). (a) The plot of the association tests of T1D patients with low T1D PRS compared to controls with low T1D PRS; (b) The plot of the association tests of all T1D patients compared to all controls.

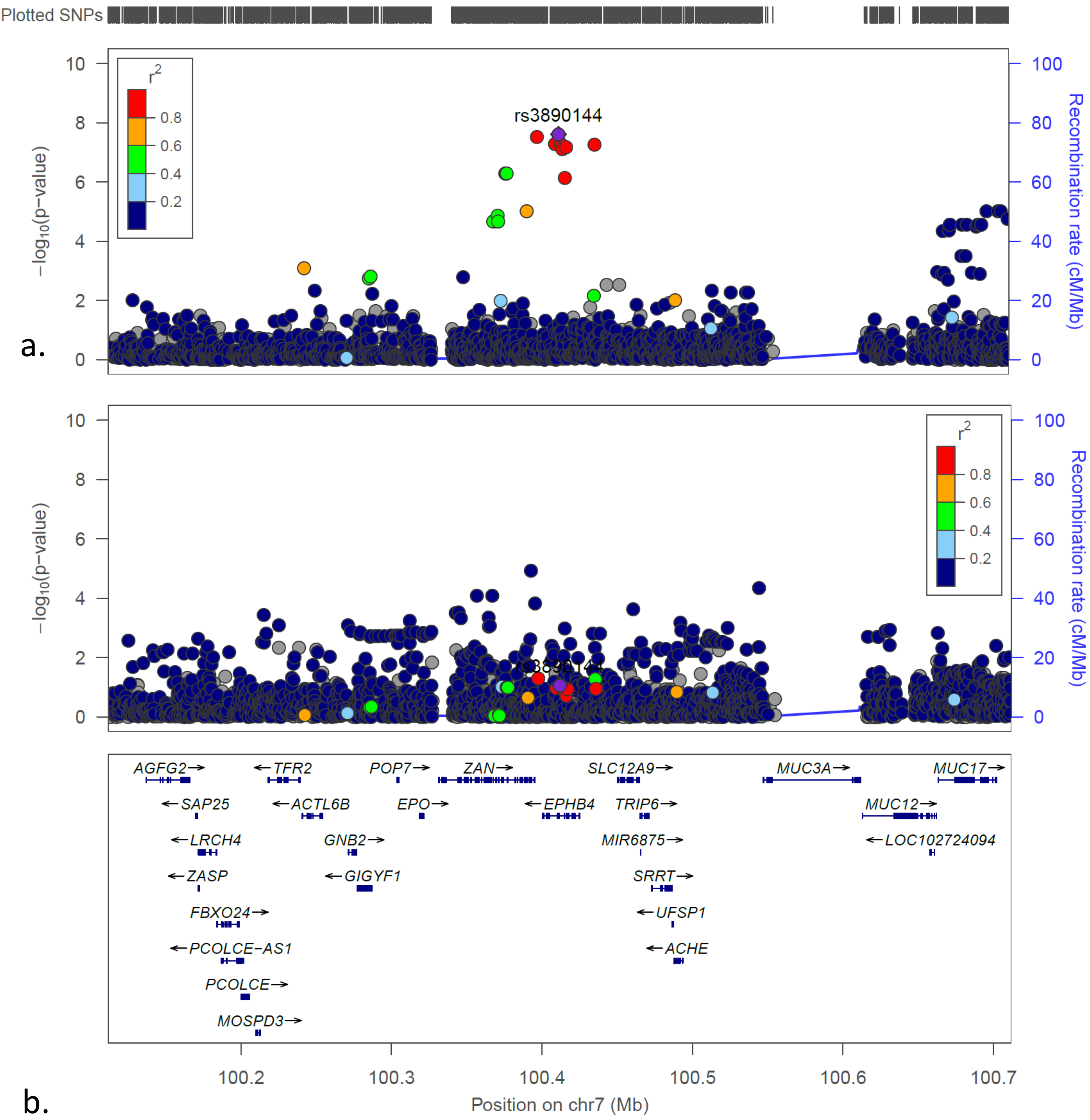

Supplementary Figure 17. The LocusZoom plots for the *EPHB4* locus (rs3890144). (a) The plot of the association tests of T1D patients with low T1D PRS compared to controls with low T1D PRS; (b) The plot of the association tests of all T1D patients compared to all controls.

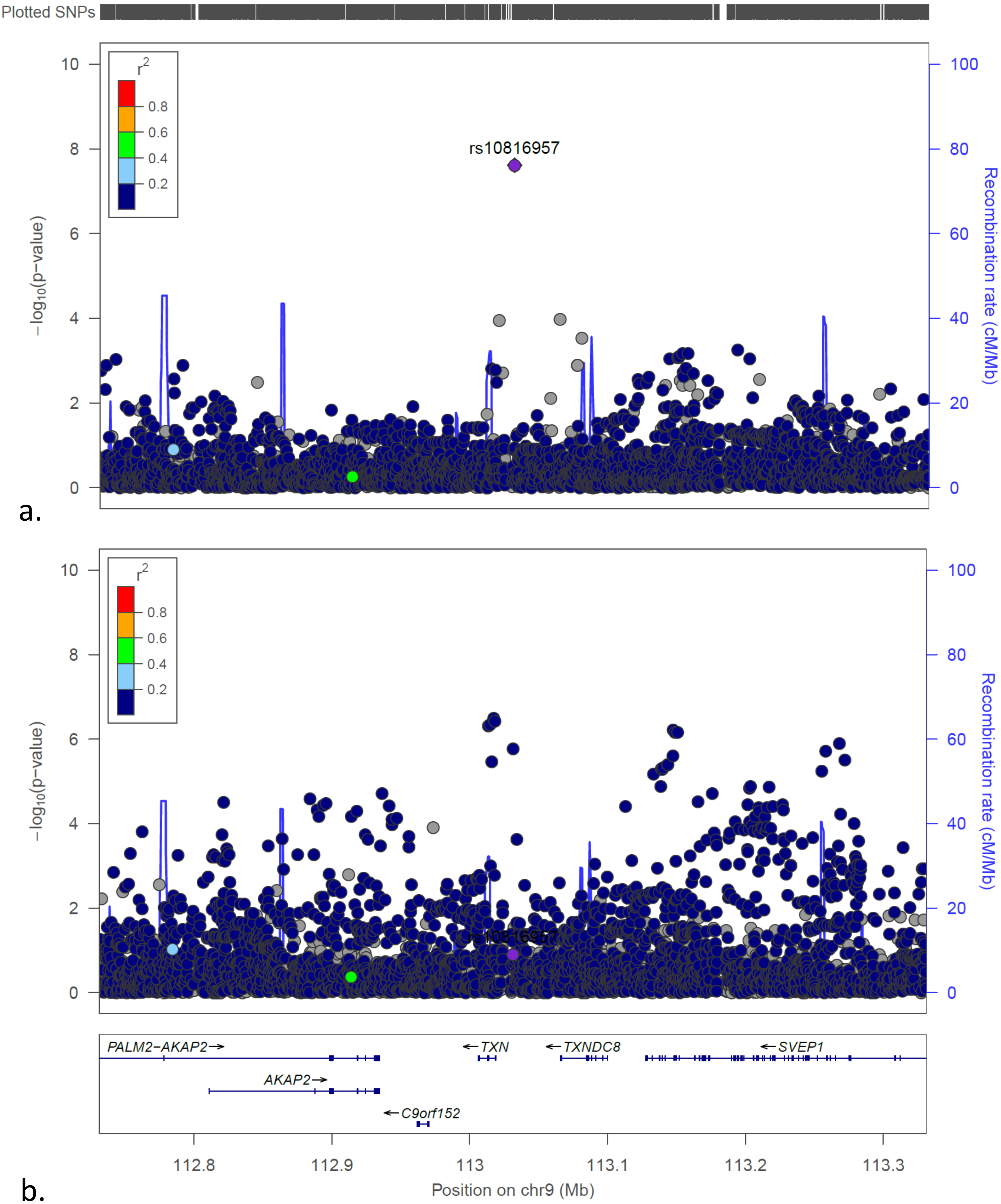

Supplementary Figure 18. The LocusZoom plots for the *TXN/TXNDC8* locus (rs10816957). (a) The plot of the association tests of T1D patients with low T1D PRS compared to controls with low T1D PRS; (b) The plot of the association tests of all T1D patients compared to all controls.

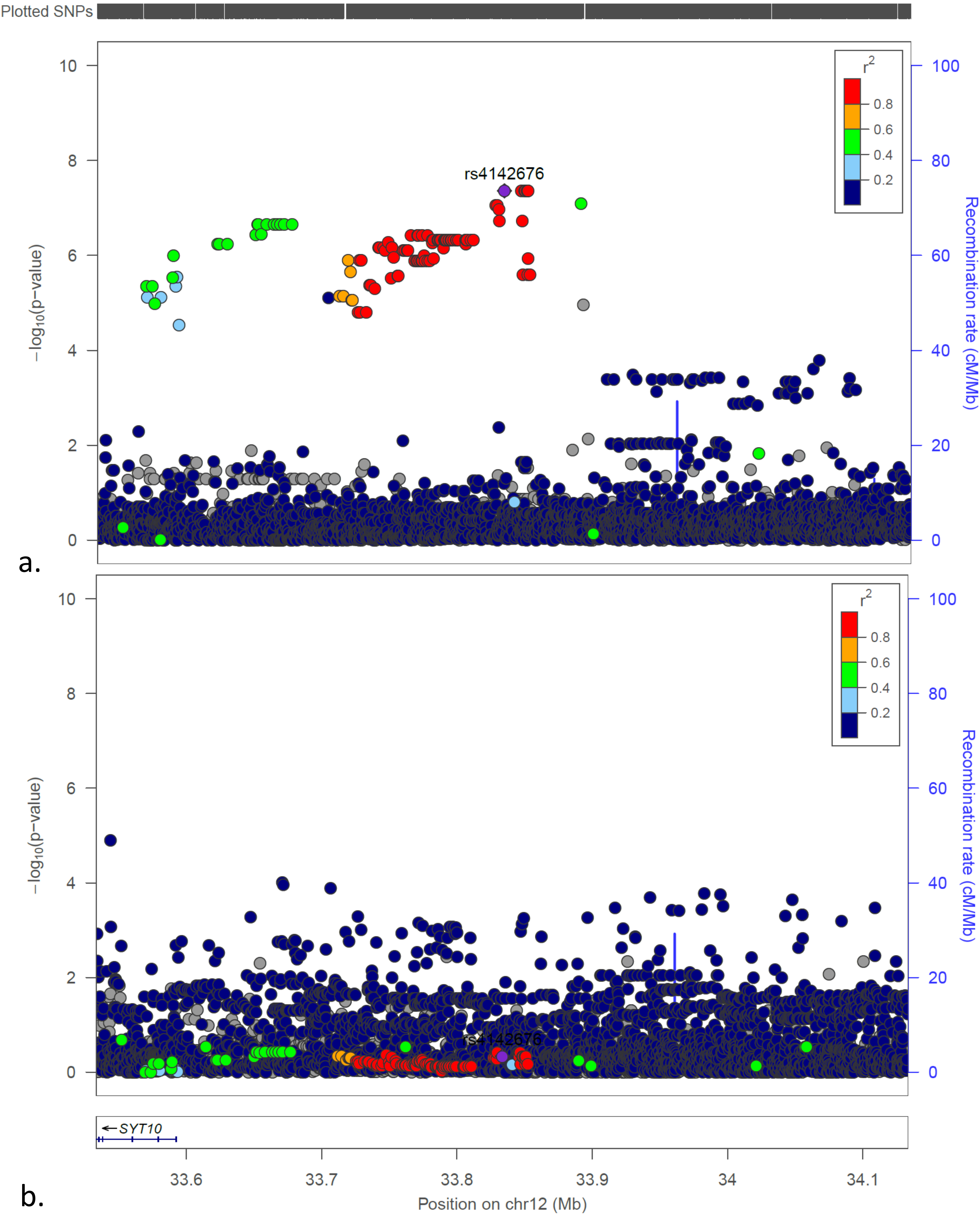

Supplementary Figure 19. The LocusZoom plots for the *SYT10/ALG10* locus (rs4142676). (a) The plot of the association tests of T1D patients with low T1D PRS compared to controls with low T1D PRS; (b) The plot of the association tests of all T1D patients compared to all controls.

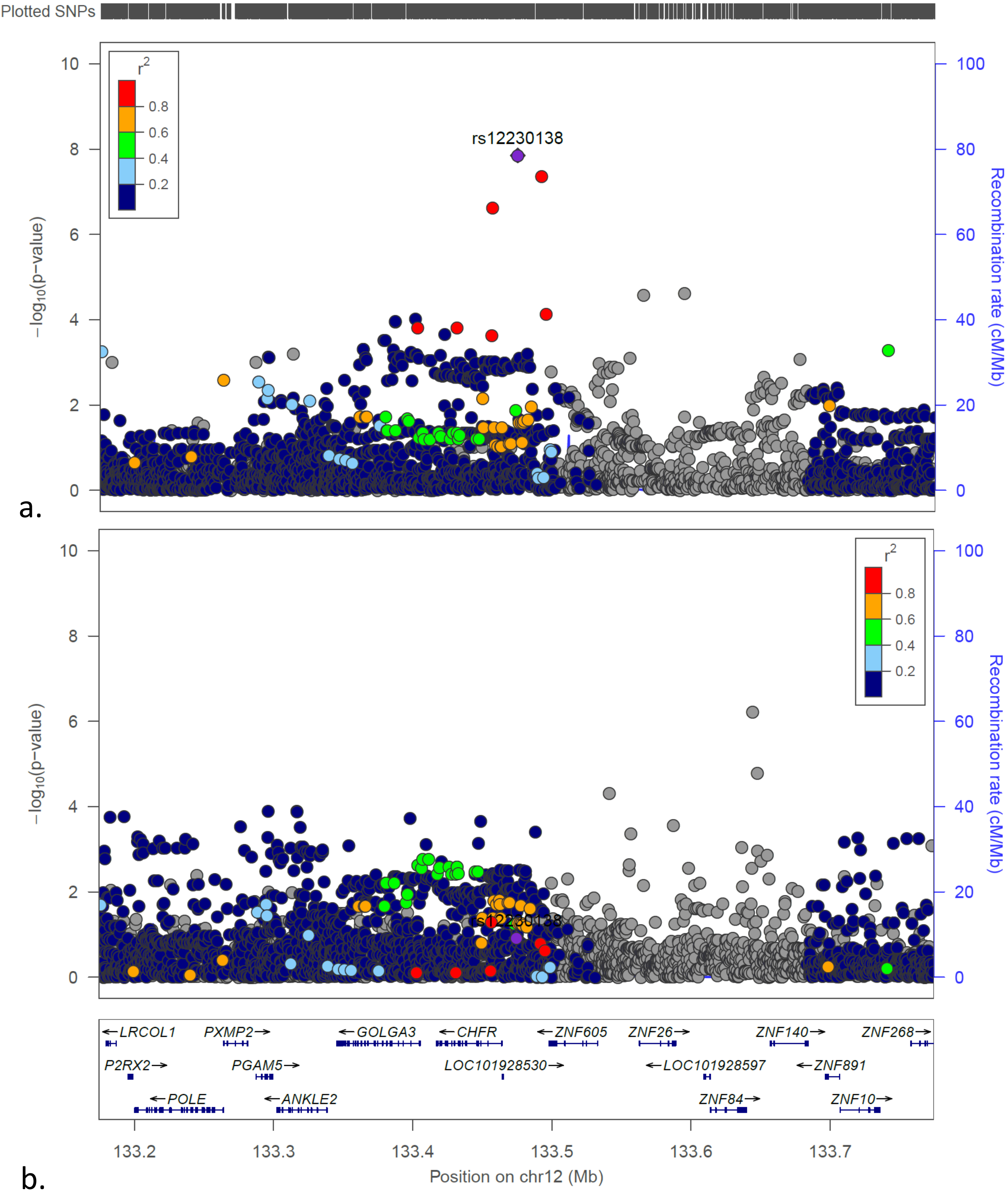

Supplementary Figure 20. The LocusZoom plots for the *LOC101928530/ZNF605* locus (rs12230138). (a) The plot of the association tests of T1D patients with low T1D PRS compared to controls with low T1D PRS; (b) The plot of the association tests of all T1D patients compared to all controls.

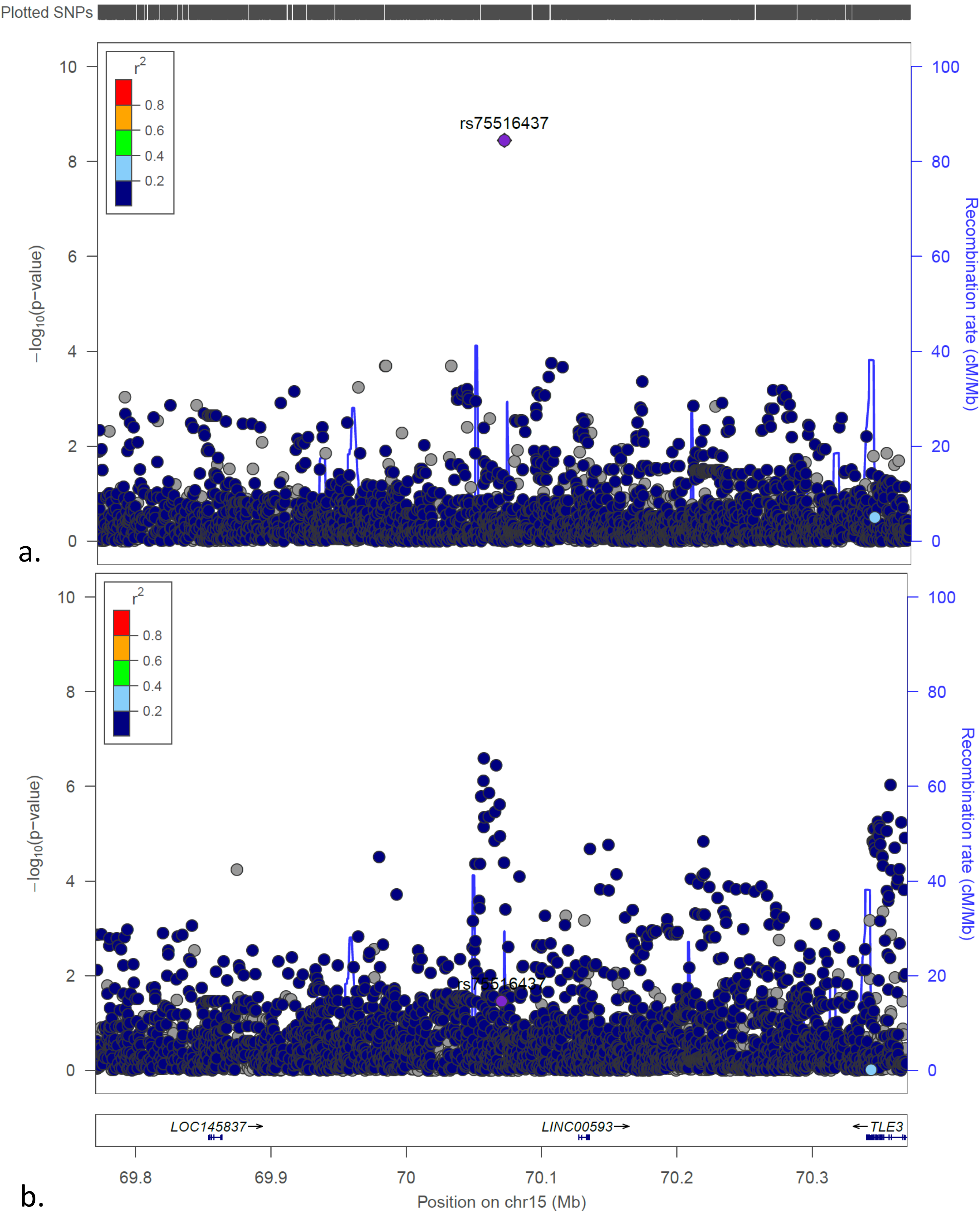

Supplementary Figure 21. The LocusZoom plots for the *PCAT29/LINC00593* locus (rs75516437). (a) The plot of the association tests of T1D patients with low T1D PRS compared to controls with low T1D PRS; (b) The plot of the association tests of all T1D patients compared to all controls.

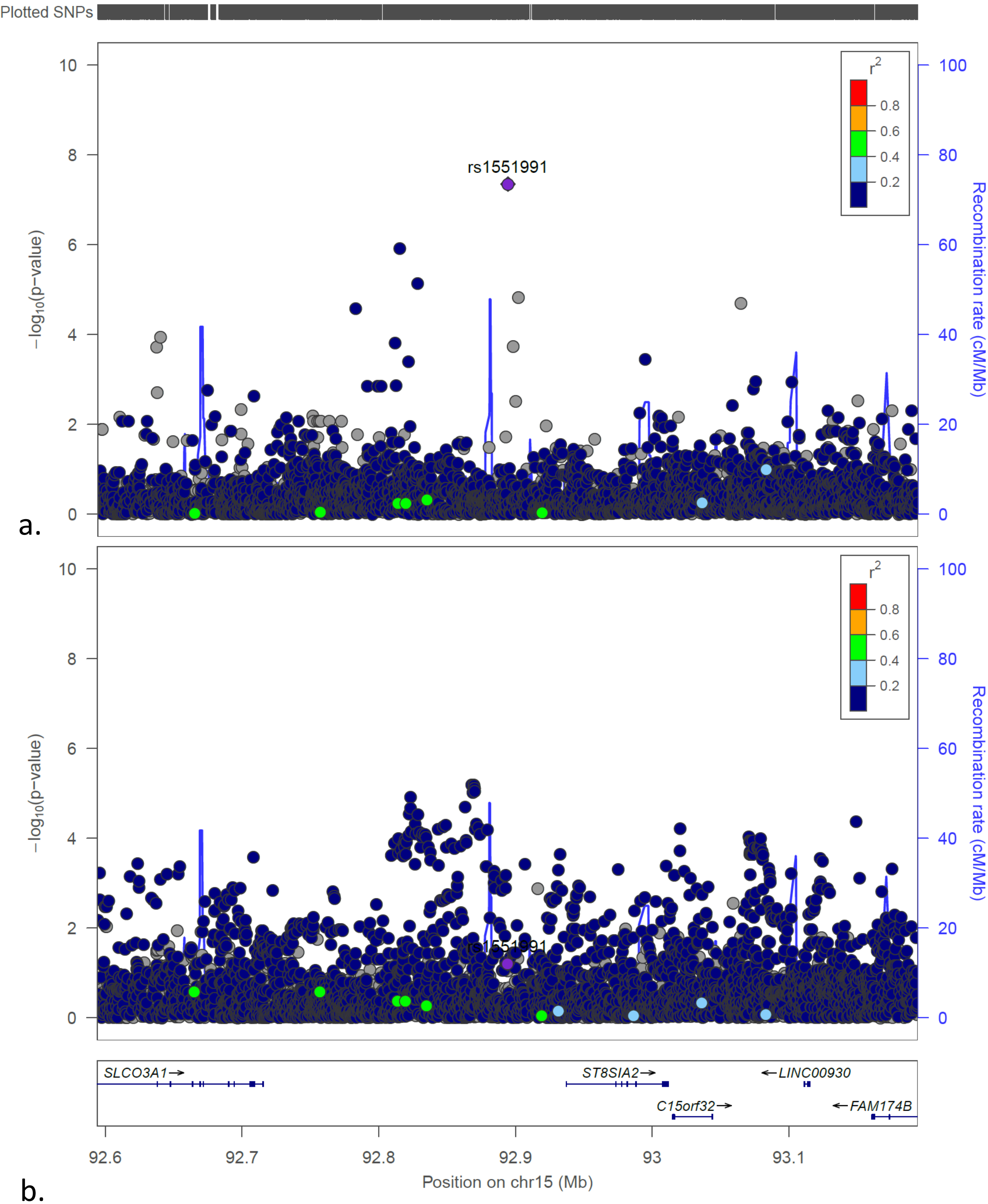

Supplementary Figure 22. The LocusZoom plots for the *SLCO3A1/ST8SIA2* locus (rs1551991). (a) The plot of the association tests of T1D patients with low T1D PRS compared to controls with low T1D PRS; (b) The plot of the association tests of all T1D patients compared to all controls.

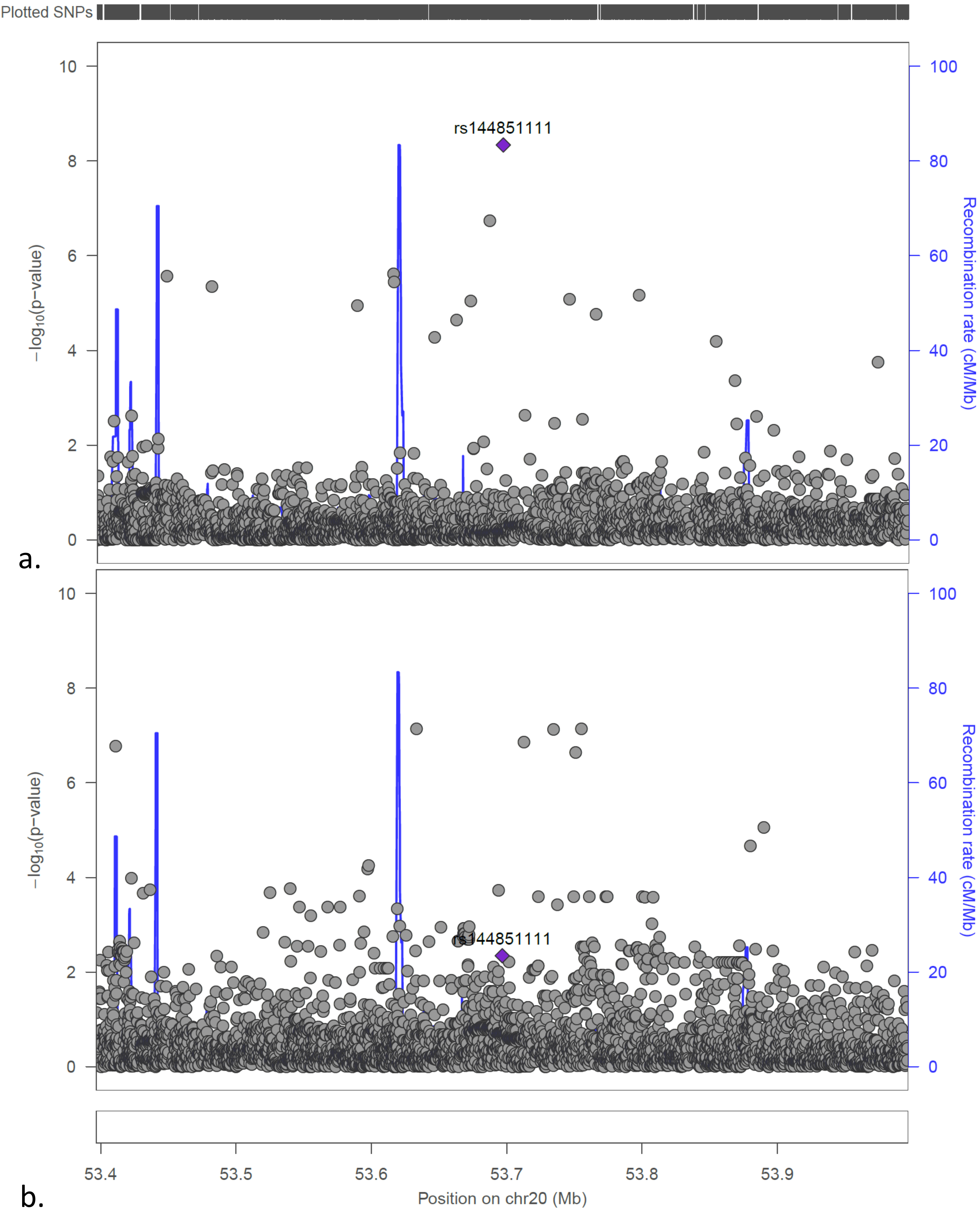

Supplementary Figure 23. The LocusZoom plots for the *DOK5/LINC01441* locus (rs144851111). (a) The plot of the association tests of T1D patients with low T1D PRS compared to controls with low T1D PRS; (b) The plot of the association tests of all T1D patients compared to all controls.

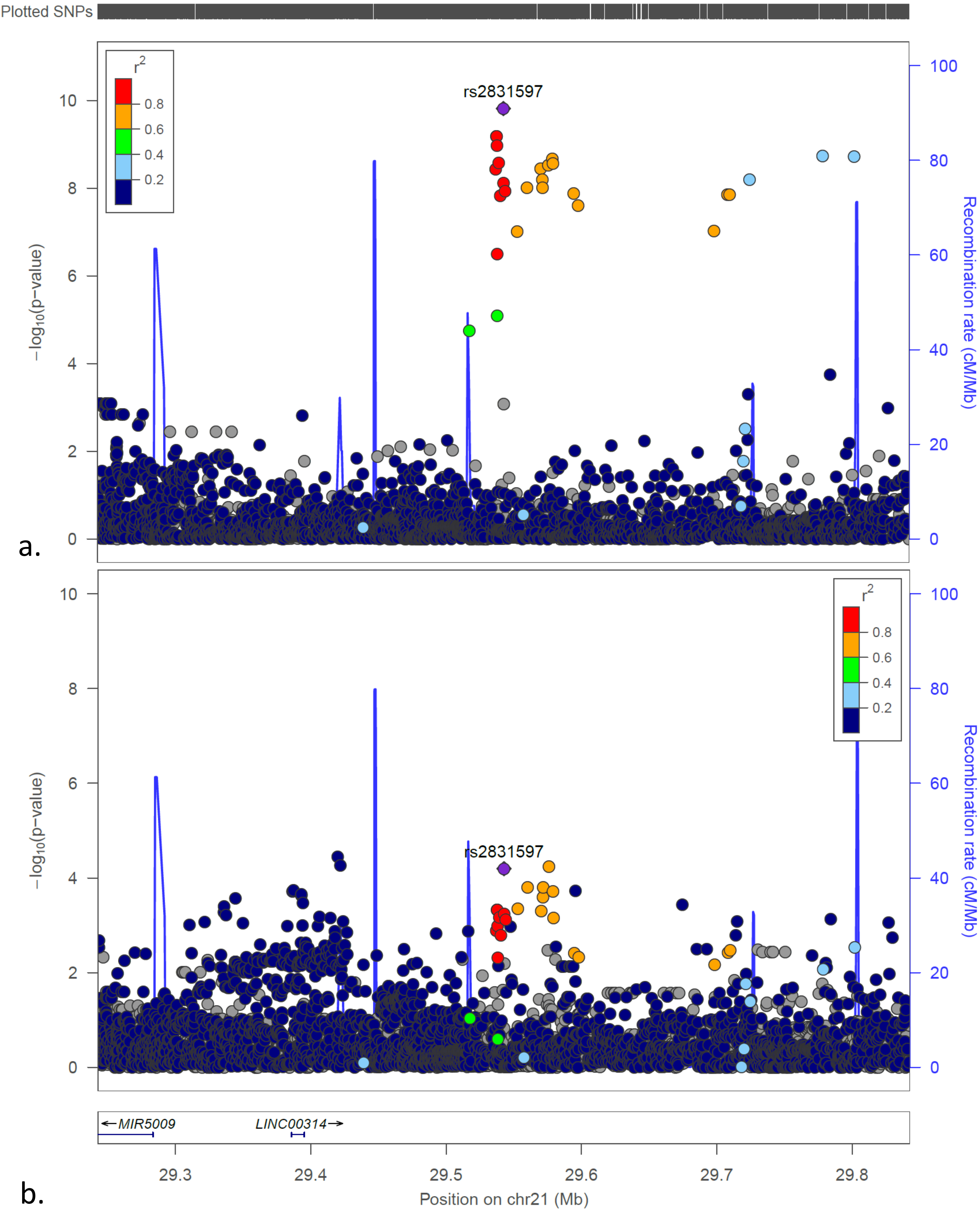

Supplementary Figure 24. The LocusZoom plots for the *LINC01695* locus (rs2831597). (a) The plot of the association tests of T1D patients with low T1D PRS compared to controls with low T1D PRS; (b) The plot of the association tests of all T1D patients compared to all controls.

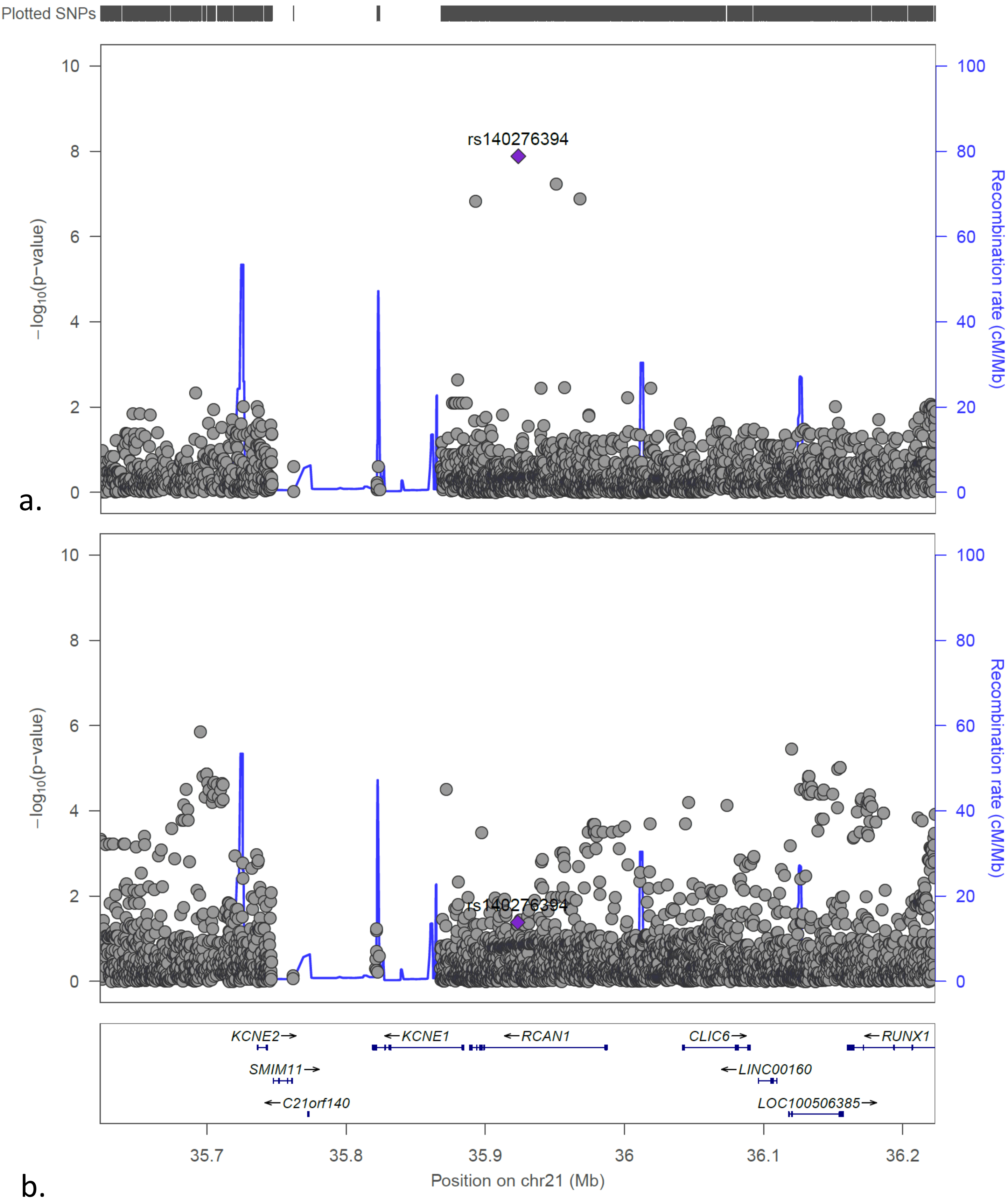

Supplementary Figure 25. The LocusZoom plots for the *RCAN1* locus (rs140276394). (a) The plot of the association tests of T1D patients with low T1D PRS compared to controls with low T1D PRS; (b) The plot of the association tests of all T1D patients compared to all controls.
